# Endophytic Entomopathogenic Fungi Shape Herbivore Behavior and Plant–Insect Interactions: Implications for Biological Control

**DOI:** 10.3390/pathogens15070735

**Published:** 2026-07-13

**Authors:** Rana H. M. Hussien, Alexandra M. Kortsinoglou, Martyn J. Wood, Vassili N. Kouvelis, Wanissa Mellikeche, Mustapha Touray, Babalwa Tembeni, Mazen Alzain, Faisal Alotaibi, Islam S. Sobhy, Zack Saud, E. Joel Loveridge, Daniel C. Eastwood, Tariq M. Butt

**Affiliations:** 1 Plant Protection Research Institute, Agricultural Research Center (ARC), Giza 12511, Egypt; 2 Natural Products BioHub, Department of Biosciences, Faculty of Science and Engineering, Swansea University, Swansea SA2 8PP, UK; wanissa.mellikeche@swansea.ac.uk (W.M.); mustapha.touray@swansea.ac.uk (M.T.); babalwa.tembeni@swansea.ac.uk (B.T.); 3 Section of Genetics and Biotechnology, Department of Biology, National and Kapodistrian University of Athens, 15771 Athens, Greece; alexkortsi@biol.uoa.gr (A.M.K.); kouvelis@biol.uoa.gr (V.N.K.); 4 Institute of Molecular Biology and Biotechnology, Foundation for Research and Technology Hellas (IMBB-FORTH), Nikolaou Plastira 100, GR-70013 Heraklion, Greece; martyn_wood@imbb.forth.gr; 5 International Centre for Advanced Mediterranean Agronomic Studies, Via Ceglie, 9-70010 Valenzano, BA, Italy; 6 Department of Biosciences, Faculty of Science and Engineering, Swansea University, Swansea SA2 8PP, UK or malzin@ut.edu.sa (M.A.); 2364417@swansea.ac.uk (F.A.); d.c.eastwood@swansea.ac.uk (D.C.E.); 7 Department of Biology, College of Science, University of Tabuk, Tabuk 23453, Saudi Arabia; 8 School of Biosciences, Cardiff University, Cardiff CF10 3AX, UK; i.sobhy@chester.ac.uk; 9 School of Natural Sciences, University of Chester, Chester CH1 4BJ, UK; 10 Department of Plant Protection, Faculty of Agriculture, Suez Canal University, Ismailia 41522, Egypt; 11 Infection and Immunity, School of Medicine, Cardiff University, Cardiff CF14 4XN, UK; saudz@cardiff.ac.uk; 12 Department of Chemistry, Faculty of Science and Engineering, Swansea University, Swansea SA2 8PP, UK; e.j.loveridge@swansea.ac.uk

**Keywords:** endophytic entomopathogenic fungi, plant physiology, pest development, fitness, survival, oviposition behavior, arthropod crop pests

## Abstract

Entomopathogenic fungi (EPF) are well established as biological control agents, but their emerging role as endophytes reveals a broader and more powerful function in crop protection. By colonizing plant tissues, endophytic entomopathogenic fungi (EEPF) create a dynamic tripartite interaction between plants, fungi, and herbivores, enabling systemic, plant-mediated pest suppression. This review synthesizes current knowledge on the behavioral and ecological responses of herbivorous arthropods to EEPF-colonized plants, with an emphasis on the mechanisms and implications for integrated pest management (IPM). Growing evidence indicates that EEPF consistently modify herbivore behavior and performance across diverse crops and insect taxa. Colonization frequently alters feeding, host selection, and oviposition, often deterring pests, although mediated responses may vary among fungal species, host plants, insect taxa, and environmental conditions. These responses are driven by EEPF-induced changes in plant chemistry, including shifts in volatile organic compounds (VOCs) and defensive metabolites. In parallel, EEPF impair insect fitness by delaying development, reducing survival, and lowering fecundity, thereby suppressing pest populations. These plant-mediated and behavioral changes extend to multitrophic interactions, potentially affecting associations with natural enemies and the transmission efficiency of some insect vectors of plant viruses. Despite rapid progress, critical gaps remain in resolving the mechanistic basis of these interactions and their stability under field conditions. Advancing the application of EEPF will require integrated approaches combining microbial ecology, chemical ecology, and insect behavioral biology. Harnessing these interactions offers a compelling pathway to reduce reliance on synthetic pesticides while enhancing the resilience and sustainability of agricultural systems.

## 1. Introduction

Entomopathogenic fungi (EPF) are key biological control agents (BCAs), contributing to sustainable agriculture and compliance with regulatory ‘green’ directives. To date, their development and application have largely focused on arthropod pest management, with over 67% of commercial products based on *Metarhizium* and *Beauveria* species [1,2]. Demand for these fungi has grown substantially, driven by regulatory measures (e.g., EU Directive 2009/128/EC; UK Pesticides National Action Plan 2025), consumer and retailer preferences for residue-free products, and environmental advocacy for biodiversity. Successful use of EPF depends on sufficient inoculum contacting the host surface under favorable environmental conditions, with the speed of kill determined by virulence and specificity determinants [3,4]. Pest control with EPF can prove challenging for various reasons. For example, EPF tend to be more host-specific than synthetic chemical insecticides, are less effective at extreme temperatures, need high humidity, and are killed quickly by UV radiation [5,6,7]. These problems are exacerbated as some pests actively avoid the fungus, potentially due to the emission of VOCs with repellent properties [4,8,9,10,11]. Despite these issues, EPF have shown significant promise as field-applied insecticides.

**Figure 1:**
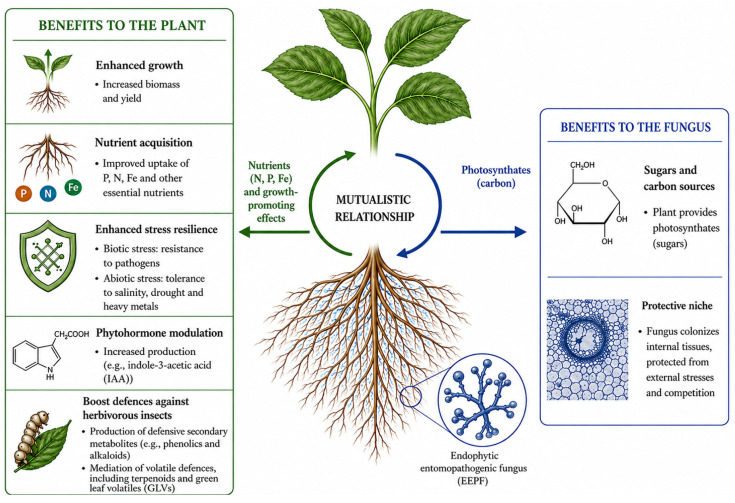
Interaction between endophytic entomopathogenic fungi (EEPF) and plants. The plant provides carbon sources and a protective niche, while the fungus enhances nutrient uptake (e.g., P, N, and Fe), promotes growth via phytohormone production (e.g., indole-3-acetic acid), and increases tolerance to biotic and abiotic stresses.

Hypocrealean EPF have been recovered from soils from around the world, with the most commonly reported genera being *Metarhizium*, *Beauveria*, *Cordyceps*, and *Lecanicillium* [5]. These fungi have had a long (>300 million years) evolutionary relationship with plants, with evidence suggesting that plant-associated lifestyles may have preceded, or co-evolved with, the diversification of insect pathogenicity in these fungi [12,13,14]. Despite acquiring insect-pathogenic traits, various EPF have retained their ancestral capacity to colonize the rhizosphere, plant roots, and other tissues endophytically and have been isolated from a diverse range of both wild and cultivated angiosperms and gymnosperms [15,16,17,18,19]. Plant–EEPF interactions are not universally beneficial. Their outcomes range from mutualistic to neutral, depending on the host plant, fungal strain, colonization level, and environmental conditions [20,21]. Nevertheless, when a mutualistic association is established, the plant provides sugars and a protective environment while the fungus transfers essential nutrients (e.g., P, N, Fe) and increases the host’s resilience to biotic (e.g., pathogens) and abiotic (e.g., salinity, drought, heavy metal) stress [18,22,23,24,25] (Figure 1). EEPF appear to stimulate plant growth through the transfer of nutrients and production of phytohormones (e.g., indole-3-acetic acid) [26,27,28]. Plants appear to recognize EEPF as symbionts, permitting entry via natural openings and direct penetration of epidermal tissues, often without causing visible disease symptoms. They then multiply at the inoculation site before translocating to other parts of the plant, with recovery usually highest close to the application site. Although natural colonization of plants by EEPF has been documented [20,29], artificial inoculation techniques are commonly employed to establish and study endophytic associations under controlled conditions. Therefore, the efficiency and extent of endophytic colonization can be influenced by the inoculation technique used. Common plant inoculation methods include seed treatment, root drenches or dips, and foliar application [30,31,32].

Studies demonstrating increased plant growth following EEPF application may result from bio-stimulatory, bio-fertilizing effects, pest-behavior modification, or a combination of all [30,33,34,35,36,37,38]. Of particular interest is the ability of EEPF to impair herbivore performance, including reduced feeding, decreased survival, delayed development, altered oviposition behavior, and diminished female fecundity in insects associated with EEPF-colonized plants [36,37,38,39,40]. In some instances, EEPF can also influence the behavior of arthropod predators and parasitoids [41,42], highlighting the importance of understanding the complex plant–fungus–insect interactions that underpin these effects. A greater understanding of the mechanismsof these multitrophic interactions will be paramount for designing effective strategies for IPM.

This review focuses on the influences that EEPF exert on some of the world’s most economically damaging arthropod pests and vectors of plant diseases. Although previous reviews have examined the endophytic lifestyle, plant growth-promoting effects, and biocontrol potential of EEPF, a comprehensive synthesis of how EEPF-mediated changes in plant physiology and chemistry influence herbivore behavior, performance, and multitrophic interactions remains lacking. Particular attention is given to the changes within host plants that subsequently influence pest behavior and performance. The review first examines the biochemical and physiological changes induced in plants by EEPF colonization, including alterations in plant secondary metabolites, fungal-derived metabolites and phytohormone signaling pathways associated with induced resistance. It then considers how these changes, especially plant VOC emissions, influence herbivore host selection, feeding behavior and oviposition decisions, as well as interactions with predators and parasitoids. Finally, the impacts of EEPF on herbivore performance, survival, and development across major feeding guilds are synthesized. Together, these sections provide an integrated overview of the mechanistic and ecological processes through which EEPF can contribute to crop protection and highlight their potential for incorporation into future IPM strategies.

## 2. Plant–EEPF Interactions

### 2.1. Plant Secondary Metabolites

**Figure 2:**
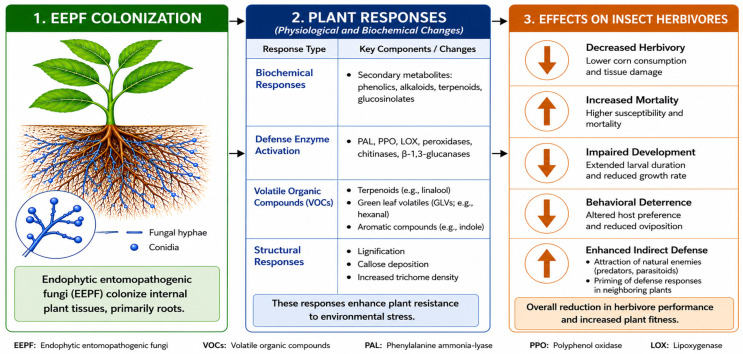
Summary of the effects of endophytic entomopathogenic fungi (EEPF) colonization on plant–insect interactions. Following plant colonization, EEPF induce biochemical, enzymatic, volatile, and structural defense responses in the host plant. These changes reduce herbivore feeding, impair insect development, increase mortality, and deter oviposition. In addition, EEPF-mediated alterations in plant volatile emissions may enhance indirect defenses by attracting natural enemies. Collectively, these responses contribute to improved plant resistance and overall fitness.

Colonization by EEPF and other endophytic fungi influences host plant metabolism, triggering physiological and biochemical responses, enhancing plant performance and resilience against biotic and abiotic stresses [20,43]. These responses include changes in primary and secondary metabolism, modulation of phytohormone signaling networks, and activation of defense-related pathways [2,44,45,46]. Metabolic reprogramming induced by EEPF may involve the upregulation or downregulation of specific metabolites, as well as the synthesis of novel tissue-specific compounds, including defense-associated metabolites such as sterols, fatty acids, terpenoids, alkaloids, phenolics, and flavonoids, together with defense-related enzymes such as peroxidases, which contribute to plant resistance against arthropod herbivores [47,48,49,50,51,52,53]. These compounds may accumulate locally or systemically throughout plant tissues or be released as volatile signals from leaves and roots. Such biochemical changes can reduce herbivore performance by deterring feeding, disrupting growth and development, lowering reproductive success, and ultimately decreasing survival and fecundity (Figure 2).

Several studies have shown that EEPF colonization can alter host plant metabolism across diverse plant systems. For example, colonization of tomato and potato plants by *Beauveria bassiana* was associated with alterations in metabolite composition, including increased levels of terpenoids and other secondary metabolites commonly implicated in plant defense against herbivores [52]. In tomato, reduced *Bemisia tabaci* infestation and damage were observed in *B. bassiana*-colonized plants, coinciding with the accumulation of several putative defense-related metabolites, including α-solanine, 5-O-caffeoylshikimic acid, and clerodendrin A [54]. Colonization of tea plants by *B. bassiana* led to a dynamic reprogramming of secondary metabolism, with an early enhancement in brassinolide and flavonoids to promote growth and enhance antioxidant capacity. As colonization continued, higher levels of catechins and epicatechins supported long-term defense and functional quality. Moreover, the elevation in chorismate indicated enhanced metabolic flux toward defense-related compounds and growth-regulating pathways, contributing to improved plant growth and stress resistance. Together, these studies demonstrate that EEPF colonization can modify host plant metabolism, although further research is needed to determine the specific roles of these metabolic changes in mediating plant growth, defense, and stress tolerance [55].

In a manner comparable to herbivore attack, EEPF commonly induce the phenylpropanoid pathway, which drives the synthesis of phenolic compounds such as phenolic acids (e.g., hydroxybenzoic and hydroxycinnamic acids), flavonoids, tannins, stilbenes, and lignans [47,56,57]. These phenolics serve multiple anti-herbivore roles, including reducing palatability, exerting direct toxicity, and interfering with digestive enzymes [50,58,59,60]. They also mitigate oxidative damage by scavenging reactive oxygen species (ROS) and stabilizing membrane lipids [57]. The defensive impact of phenolics was demonstrated by Darsouie et al. [51], who recorded increased phenolic content in *Beta vulgaris* leaves colonized by *B. bassiana* or *Beauveria varroae*. Colonization inhibited *Spodoptera littoralis* larval development by interacting with and reducing gut protease and lipase activity. Similarly, *B. bassiana* colonization induced resistance in tomato by activating the isoflavonoid pathway. This triggered the upregulation of the *SlHIDH* gene, resulting in a 3.5-fold accumulation of the isoflavonoid genistein. This metabolite acts as a deterrent, impairing the reproductive physiology of the whitefly *B. tabaci* through disruption of ovarian development and reproduction-related gene expression [61]. Although *B. bassiana* and *Metarhizium anisopliae* reduced *Aphis gossypii* populations on cucumber plants by 32–35%, and *B. bassiana* induced the highest total phenolic content, these phenolic compounds alone did not fully explain the observed pest suppression [62].

Importantly, EEPF not only stimulate the direct production of defensive metabolites in the absence of herbivory, but also prime plant defense mechanisms. For example, *B. bassiana* inoculation in tomato upregulated key defense-related genes, including *PIN2*, *PR2*, *PAL*, and *MPK3*, whereas *B. tabaci* feeding alone induced only mild gene expression. However, when fungal colonization and herbivore feeding occurred simultaneously, nine defense-related genes were activated, five of which (*PAL*, *PPO*, *PIN2*, *PR2*, and *PR1*) are closely associated with phenolic compound biosynthesis. These transcriptional changes were accompanied by increased phenolic accumulation and enhanced phenylalanine ammonia-lyase (PAL) activity in EEPF-colonized plants [63]. Consistent with these findings, increased levels of phenolics, antioxidant compounds, and other secondary metabolites have been reported in onion, potato, and *Helichrysum* plants colonized by *B. bassiana* [52,64,65]. These studies suggest that EEPF can function as biotic elicitors of plant defense responses, enhancing defense-related metabolic and transcriptional pathways without causing apparent damage to the host plant. This highlights their potential for improving plant resilience and supporting sustainable pest management strategies.

### 2.2. EEPF-Derived Secondary Metabolites

Many endophytic fungal BCAs, including EEPF and mycoparasites, are known to secrete an array of secondary metabolites, some functioning as potent toxins, while others exhibit antifeedant, antimicrobial, or deterrent activity [4,66,67,68,69]. These bioactive compounds may act directly or synergistically with plant metabolites to protect host plants from a broad spectrum of herbivores and microbial pathogens. Insects often avoid plants coated in or emitting these metabolites, and contact with or ingestion of them can result in physiological disruption or mortality [70,71,72,73]. Despite the diversity and potency of these compounds in vitro, there is limited evidence that EEPF produce secondary metabolites in plants at concentrations that pose a risk to humans or livestock [67,74]. Most studies report only transient or very low levels of these compounds in plants, or infer their presence based on indirect observations [75,76,77]. This limitation is primarily attributed to the localized and transient nature of EEPF growth within plant tissues, which restricts fungal biomass and, consequently, metabolite production [32,78,79]. Consequently, any negative effects observed in herbivores feeding on EEPF-colonized plants are more plausibly explained by EEPF-triggered plant defense responses or broader changes in plant metabolism, rather than by direct toxicity from fungal metabolites.

Nevertheless, some studies suggest that EEPF may still influence herbivore performance through the localized accumulation of secondary metabolites within host tissues. For instance, Jaber and Araj [80] observed an increase in winged morphs of *Myzus persicae* on colonized sweet pepper, suggesting a metabolite-driven dispersal response. Interestingly, Golo et al. [75] detected destruxins A, B, and E in cowpea plants colonized by *Metarhizium robertsii*, although these compounds were not found in colonized cucumber plants. Furthermore, destruxins were not detected in either cowpea or cucumber plants colonized by *M. acridum*, indicating complex and specific species-specific interactions that result in a high degree of variability in metabolite production.

Importantly, available evidence indicates that EEPF exert minimal direct toxicity toward non-target organisms, with experimental studies demonstrating no significant negative effects on key BCAs, including predators such as *Phytoseiulus persimilis* and *Chrysoperla carnea*, while vertebrate safety assessments report negligible pathogenicity under typical exposure conditions [78,81,82].

Insects succumbing to EPF infection will sporulate if conditions are appropriate, but this is rarely reported for insects feeding on plants colonized by EEPF. Insect mortality following feeding on EEPF-colonized plants has been suggested to result from reduce nutritional quality, noxious secondary plant metabolites, and other stress-causing agents, rather than direct fungal infection or fungal metabolite toxicity [78]. In many studies, fungal propagules were never recovered even when dead insects were incubated under optimal laboratory conditions, despite EPF re-isolated from inoculated plants being virulent toward larvae [83,84,85,86].

### 2.3. EEPF-Mediated VOC Defenses

#### 2.3.1. EEPF Influence on Plant Volatile-Mediated Insect Defense

**Figure 3:**
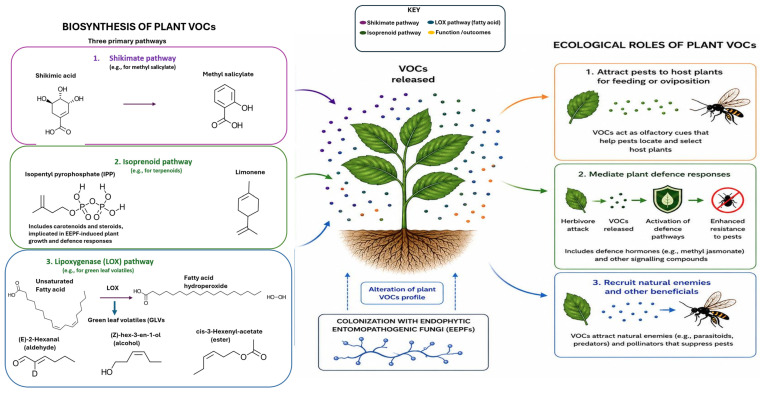
Overview of plant volatile organic compound (VOC) biosynthesis and its ecological roles. VOCs are produced via the shikimic acid, isoprenoid, and fatty acid/lipoxygenase (LOX) pathways. Colonization by endophytic entomopathogenic fungi (EEPF) alters VOC profiles, influencing plant–insect interactions by reducing herbivore attraction, enhancing plant defense responses, and promoting the recruitment of natural enemies.

Plants emit a diverse array of VOCs that play central roles in plant–arthropod interactions, influencing host location, feeding behavior, oviposition, and indirect defense responses (Figure 2 and Figure 3) [87,88]. Based on their chemical structures and biosynthetic origins, plant VOCs are generally classified into four major groups: terpenoids, fatty acid-derived green leaf volatiles (GLVs), phenylpropanoids/benzenoids, and nitrogen- or sulfur-containing compounds [89,90]. Their biosynthesis is primarily regulated by three metabolic pathways: the shikimate pathway, which produces aromatic compounds such as methyl salicylate; the isoprenoid pathway, responsible for terpenoid synthesis; and the fatty acid/lipoxygenase pathway, which generates GLVs [90,91].

Among these, terpenoids and other isoprenoid-derived compounds play important roles in plant growth, stress tolerance, and induced defense responses, including those mediated by EEPF [49]. Fatty acid derivatives originate from unsaturated fatty acids and include signaling molecules such as methyl jasmonate, a key phytohormone involved in plant defense regulation and stress signaling [63,92]. GLVs are rapidly released following mechanical injury or herbivore attack and are responsible for the characteristic odor of damaged foliage. These compounds mainly consist of six-carbon aldehydes (e.g., (E)-2-hexenal), alcohols (e.g., (Z)-3-hexenol), and esters, including acetates, butyrates, and benzoates (Figure 3) [93,94].

Insects are not only able to perceive but also distinguish between the relative quantities of plant VOCs, which helps them select suitable host plants [95]. EEPF can alter plant volatile composition or the ratios between certain volatiles [96]. For instance, inoculation of sweet pepper plants with *Akanthomyces muscarius* (=*Lecanicillium lecanii*) reduced β-pinene and indole emissions [97]. Increased emissions of 2-ethyl-1-hexanol and (Z)-3-hexen-1-ol, along with reduced emissions of β-caryophyllene, naphthalene, and α-pinene, were observed following *B. bassiana* colonization of maize [39]. Similarly, melon and cotton plants colonized by *B. bassiana* emitted volatile blends that differed from those of uncolonized control plants [98]. The identity of fungal species and strain, plant cultivar, and their interactions with the host plant influences plant VOC emission profiles. Indeed, large differences in biosynthetic gene repertoire have already been noted between strains of the same EPF species, suggesting that the outcomes of plant–fungal interactions may exhibit some degree of specificity [99]. Since plant volatiles are key mediators of arthropod–plant interactions, changes in VOC composition resulting from EEPF colonization may influence plant–insect interactions, including feeding and oviposition behavior, and the chemotactic responses of herbivores and their natural enemies [42,46,97,100,101,102,103]. Alterations in plant volatile emissions are not exclusive to EEPF and have also been observed following colonization by other endophytic fungi. For example, maize plants inoculated with *Trichoderma atroviride* exhibited decreased emissions of β-caryophyllene and α-humulene [104].

#### 2.3.2. Volatiles Emitted from Plants in Response to Insects and EEPF

Plants respond to arthropod herbivory through a wide range of defense mechanisms, among which the emission of VOCs represents one of the most rapid and dynamic responses [105]. These herbivore-induced plant volatiles (HIPVs) play key ecological roles, including attracting natural enemies of herbivores, signaling to neighboring plants, and coordinating systemic defense responses within the emitting plant [106,107,108]. Although simultaneous herbivory by multiple species can alter VOC composition, the overall attraction of natural enemies is generally maintained across diverse VOC blends [109].

In agroecosystems, these plant-derived VOCs have been exploited to improve pest management. For example, intercropping systems can disrupt host plant recognition by masking crop VOCs with those of non-host species, while synthetic HIPVs and genetically engineered crops with enhanced volatile emissions have been shown to attract beneficial arthropods or deter herbivores [110,111,112].

While HIPV production is a typical plant response to herbivory, VOC emissions are also influenced by endophytic colonization by EEPF. It has been demonstrated that various plants, when colonized by EEPF, release distinct VOC blends in the presence and absence of insect herbivory. For instance, *Phaseolus vulgaris* plants colonized by *Hypocrea (=Trichoderma) lixii* or *B. bassiana* produce VOC blends including terpenes such as (E)-β-ocimene, (E)-caryophyllene, and α-cedrene, as well as alkanes such as heneicosane and tridecane, many of which have known roles in plant defense against pathogens and insect herbivores [98,113]. Cotton plants predominantly released a monoterpene (i.e., myrcene) and sesquiterpenes (e.g., β-caryophyllene, α-humulene), while melon plants produced aldehydes, alcohols, acids, acetates, and their derivatives. In *B. bassiana*-colonized cotton, increased emission of compounds like α-pinene and 1H-indole was observed, both of which are associated with herbivore attack and known to attract natural enemies [98].

Some VOCs, such as indole, illustrate the overlap between plant-derived and EEPF-modulated responses. While indole is a well-established HIPV produced during herbivory, its emission can be amplified or altered by fungal colonization. For example, *A. muscarius*-inoculated sweet pepper plants released higher levels of indole and β-pinene, influencing aphid behavior, with electrophysiological assays confirming strong antennal responses to indole [97,114]. Mechanistically, indole functions as a priming signal; in tea plants, it activates early defense signaling pathways (e.g., Ca^2+^ influx and MAPK cascades), enhances jasmonate biosynthesis, and upregulates defense-related genes, ultimately increasing resistance to herbivores [115].

In addition to such shared compounds, certain VOCs have been reported in EEPF–plant–herbivore systems. Benzaldehyde, a widespread VOC emitted by both plants and insects, is multifunctional, acting as a sex, aggregation, and alarm pheromone, as well as a defensive secretion [116,117,118]. It was among the compounds reported in caterpillar-infested *B. bassiana*-colonized cotton, while 2-ethylhexyl nonyl sulfite was associated with melon plants infested by cotton aphids [98]. Similarly, the compound (2Z,13E)-octadeca-2,13-dien-1-ol found in caterpillar-infested and endophytically colonized wild cotton plants has been reported as an insect sex pheromone [119,120]. Collectively, these studies indicate that EEPF colonization may modify both the composition and abundance of HIPVs. Notably, terpenoids and other defense-associated volatiles appear among the most frequently reported EEPF-responsive compounds, suggesting a potential role in mediating indirect plant defense.

#### 2.3.3. Volatile-Mediated Modulation of Insect Feeding and Oviposition in the Presence of EEPF

Fungal endophytes may induce local or systemic changes in plant volatiles, which play a crucial role in influencing insect and mite feeding preferences [101,121]. For example, *M. persicae* preferred sweet pepper, *Capsicum annuum*, inoculated with *A. muscarius*, over uninoculated control. This preference was attributed to the increased emission of β-pinene, indole, and terpinolene caused by *A. muscarius* colonization [97]. In contrast, choice assays revealed that all developmental stages of *Thrips tabaci* preferred endophyte-free onion plants to endophytically colonized plants [122]. Similarly, Richmond [123] reported that second instar *Parapediasia teterrella* Zincken strongly preferred endophyte-free plants over endophyte-colonized plants.

EEPF elicit changes in plant VOCs, which are known to affect oviposition behavior in a broad range of insects (Figure 3). Pec et al. [102] found that sugarcane inoculated with *M. robertsii* increased salicylic acid (SA) and jasmonic acid (JA) content but suppressed the emission of plant volatiles, leading to a reduced oviposition preference by the sugarcane borer, *Diatraea saccharalis*. Tomato plants treated with *M. robertsii*, either alone or combined with *Bacillus amyloliquefaciens*, exhibited reduced *Tuta absoluta* oviposition compared to untreated controls [124]. Similarly, gravid *T. absoluta* females preferred to oviposit on EEPF-free plants over those inoculated with *M. anisopliae* or *B. bassiana* [125,126]. These effects are likely linked to changes in tomato VOC emissions, as previous studies have shown that larval feeding damage or exogenous application of methyl jasmonate can reduce insect oviposition through VOC-mediated signaling [124,127]. Munawar et al. [96] also found that adult *Phthorimaea (Tuta) absoluta* were less attracted to *Cordyceps fumosorosea*-treated plants, apparently repelled by the EPF-induced VOCs (E)-β-caryophyllene, β-phellandrene, and α-phellandrene, whose production corresponded with enhanced JA and SA production.

While there are many examples of EEPF making host plants less attractive for oviposition (Table 1, Table 2 and Table 3), contrasting responses have also been observed, highlighting that variation in host selection and oviposition responses may arise from differences in fungal species, host plant genotype, and the specific physiological changes induced in colonized plants. For instance, *Trialeurodes vaporariorum* preferred to probe tomato plants infected by *Acremonium strictum*. However, the nymphs suffered high mortality [128]. The author proposed that this reduction in whitefly performance resulted from changes in plant nutritional quality and the induction of systemic plant defenses. Similarly, gravid females of *Helicoverpa Armigera* preferred to lay eggs on tomato plants inoculated with *A. strictum* [100], but the offspring suffered from reduced growth rate, high mortality and low fecundity [129]. These findings suggest that endophyte-induced changes in plant chemistry, including alterations in volatile emission profiles, can influence herbivore host selection. Likewise, Cotes et al. [130] found that cabbage inoculated with *M. brunneum* Met52 produced higher levels of JA, (+)-7-iso-jasmonoyl-l-isoleucine, and SA in certain parts of the host plant, which increased the landing and oviposition behavior of cabbage root fly *Delia radicum* females. These studies indicate that EEPF-mediated changes in plant volatile emissions can alter herbivore host-selection and oviposition behavior. However, the direction of these responses is not uniform. While many studies report reduced attraction and oviposition on colonized plants, others demonstrate increased host preference despite subsequent reductions in herbivore performance. This variability suggests that insect behavioral responses are influenced not only by EEPF-induced changes in volatile profiles but also by species-specific differences in insect sensory ecology, host plant traits, and the physiological consequences of endophytic colonization.

### 2.4. EEPF-Mediated Phytohormone Signaling

**Figure 4:**
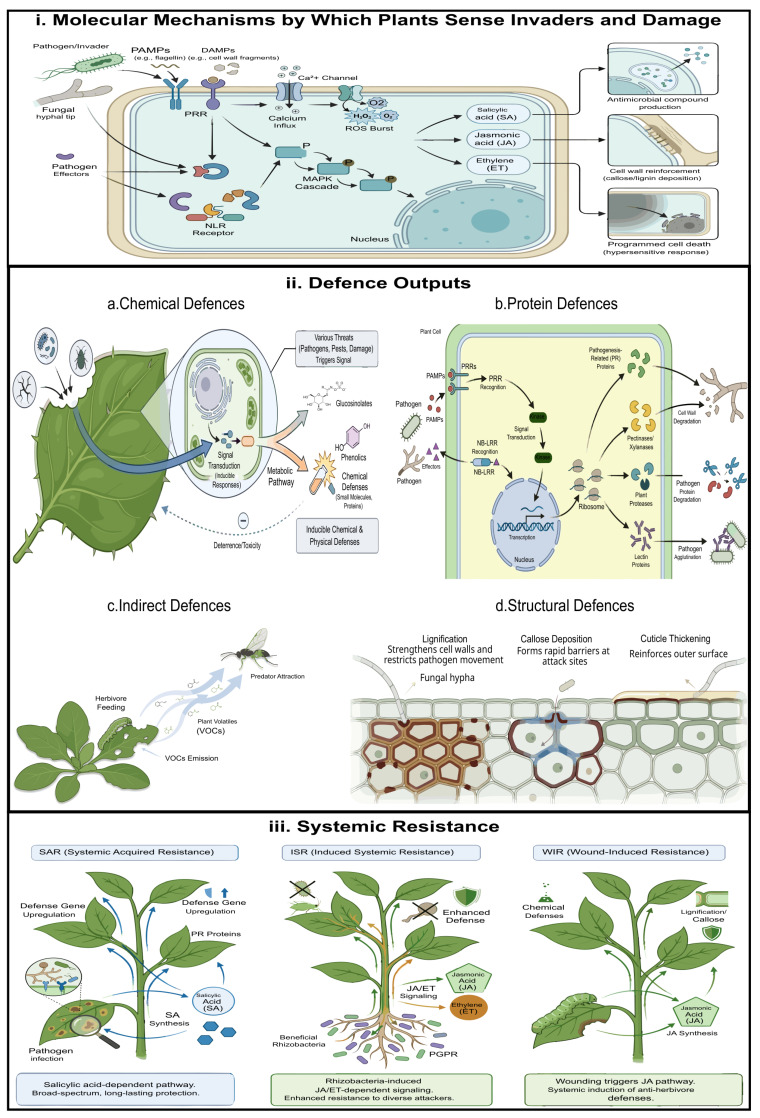
Molecular architecture of plant innate immunity, defense execution, and systemic resistance cascades. (**i**). Schematic representation of the two-tiered plant immune system showing pathogen detection and early intracellular signaling transduction. Extracellular Pattern Recognition Receptors (PRRs) detect broad-spectrum Pathogen- or Damage-Associated Molecular Patterns (PAMPs/DAMPs) to initiate Pattern-Triggered Immunity (PTI). Conversely, intracellular Nucleotide-binding Leucine-rich Repeat (NLR) receptors recognize race-specific pathogen effectors, initiating Effector-Triggered Immunity (ETI). Receptor activation rapidly induces cytosolic calcium Ca^2+^ influx, apoplastic reactive oxygen species (ROS) production, and Mitogen-Activated Protein Kinase (MAPK) cascade activation. These early events drive transcriptional reprogramming across distinct phytohormone pathways, predominantly mediated by Salicylic Acid (SA) during biotrophic invasion, or Jasmonic Acid (JA) and Ethylene (ET) during necrotrophic and herbivore attack. (**ii**). Structural and biochemical execution of localized defense responses. Downstream targets include physical reinforcements (callose deposition and cell wall lignification), direct chemical and protein-based defenses (antimicrobial phytoalexins and proteinase inhibitors), and indirect defenses via the emission of volatile organic compounds (VOCs) to attract herbivore natural predators. (**iii**). Long-distance systemic immunity networks. Localized biotic stress activates mobile signals that prime distal tissues, resulting in salicylic acid (SA)-associated Systemic Acquired Resistance (SAR), jasmonic acid (JA)/ethylene (ET)-associated Induced Systemic Resistance (ISR), and wound-induced resistance (WIR). Although illustrated separately for clarity, these pathways are highly interconnected and exhibit extensive crosstalk among SA-, JA-, and ET-mediated signaling.

Plant signaling and phytohormone responses to attack from herbivores and microbial pathogens have been extensively reviewed [131,132,133]. Comparatively little is known about plant responses to beneficial EEPF. Figure 4 provides a simplified overview of the complex signaling events in EEPF–insect–plant interactions.

Plants possess constitutive defenses, which are permanent (e.g., physical and chemical barriers), and induced defenses, which are activated by biotic (e.g., pest, pathogen) and abiotic (environmental) stresses [134,135]. Interactions between plants and beneficial microbes, including EEPF, trigger induced resistance, which is categorized into two main types: systemic acquired resistance (SAR) in response to plant pathogens and induced systemic resistance (ISR) triggered by beneficial rhizosphere microbes [136]. Unlike vertebrate adaptive immunity, SAR is broad-spectrum, with no specificity to the initial infection. An avirulent pathogen causing local programmed cell death can induce SAR through the generation of mobile signals, accumulation of the defense hormone SA, and secretion of antimicrobial pathogenesis-related (PR) proteins (Figure 4). Consequently, the rest of the plant can be protected from secondary infection for a period of weeks to months.

Plant resistance effects may also be inherited on a multigenerational level, either genetic or as a consequence of vertical transmission of the EEPF. Quesada-Moraga et al. [137,138] demonstrated that *B. bassiana* applied to opium poppy, *Papaver somniferum*, plants was vertically transmitted through seeds, being detected in up to 50% of seeds and 25% of progeny plants, which subsequently showed resistance against the stem gall wasp *Iraella luteipes. Beauveria bassiana* has been detected in seedlings of Monterey Pine, *Pinus radiata*, which were not previously exposed to the fungus, indicating successful vertical transmission from the parent tree [139]. However, Russo et al. [140] found no vertical transmission of *B. bassiana* in corn. Although not widespread, vertical transmission has also been documented in non-EPF such as *Alternaria alternata* and *Cladosporium sphaerospermum* across multiple plant species, suggesting that this phenomenon may be more common than previously thought [141]. While these fungi are not entomopathogens, their transmission patterns imply that EEPF may also persist across generations and continue to induce plant defenses in progeny. SAR can even be passed on to progeny through epigenetic regulation, specifically hypomethylation and histone acetylation, which means that changes to gene regulatory mechanisms can be inherited, leading to increased resistance to pathogens in the next generation and potentially providing a form of “plant memory” of disease stress [142,143]. Therefore, it is plausible that vertically transmitted EEPF could contribute to transgenerational defense responses, although direct experimental evidence remains limited and warrants further investigation.

EEPF enhance plant immunity by modulating systemic defense responses, primarily through induced systemic resistance (ISR) and, in some pathosystems, systemic acquired resistance (SAR). Both mechanisms activate defense-related pathways, leading to the accumulation of reactive oxygen species (ROS), callose, and other antimicrobial compounds [43,144]. Local defense responses are initiated at the site of pathogen attack, whereas SAR represents a systemic and long-lasting state of enhanced resistance that develops following local infection and provides broad-spectrum protection against subsequent pathogen challenges [142]. SAR is primarily mediated by salicylic acid (SA)-dependent signaling and is characterized by the coordinated activation of pathogenesis-related (PR) genes, including PR1, PR2, and PR5, as well as the transmission of long-distance immune signals to distal plant tissues [142]. In contrast, ISR is typically triggered by beneficial microorganisms, including EEPF, and is predominantly regulated through jasmonic acid (JA) and ethylene (ET)-dependent signaling pathways [145], often without substantial accumulation of PR proteins, although SA-dependent ISR has been reported in some cases [136]. Rather than constitutively activating defenses, both SAR and ISR establish a primed state that enables plants to respond more rapidly and robustly to subsequent challenge [146,147,148]. The extensive crosstalk among SA, JA, and ET signaling pathways allows plants to fine-tune defense responses according to the nature of the threat, where the regulation of phytohormone gene expression acts as a defensive strategy against various stresses and facilitates the successful establishment of symbioses [149,150].

While JA and ET can act synergistically to enhance resistance against necrotrophic pathogens and wounding, they may also interact antagonistically with other stress-response pathways [151,152,153]. Increasing evidence indicates that beneficial microbe-induced resistance frequently involves coordinated activation of SA- and JA/ET-dependent pathways. For example, *Trichoderma longibrachiatum* H9 enhanced cucumber growth and reduced *Botrytis cinerea* infection through the activation of both SA- and JA/ET-mediated defenses, and similar effects have been reported for other beneficial microorganisms that stimulate the accumulation of these defense-related phytohormones [154]. Similarly, endophytic colonization by *B. bassiana* and *Metarhizium* spp. has been shown to activate SA, JA-, and ET-associated defense pathways, resulting in enhanced resistance against insect herbivores and plant pathogens [53,155,156].

Induced defense responses are often mediated by phytohormone pathways, which also regulate essential functions such as growth and are broadly conserved across plant taxa [157,158]. Not surprisingly, EEPF are known to stimulate plant growth and increase resilience to environmental and biotic (herbivores, pathogens) stressors [159]. Farrokhzadeh et al. [160] showed that cotton plants inoculated with conidia of *B. bassiana* GHA triggered broad-spectrum systemic resistance through coordinated activation of multiple hormonal pathways. These researchers demonstrated that live conidia strongly upregulated four genes representing key hormonal pathways, notably ERF1 and MPK2 (ethylene), WRKY (salicylic acid), and JAZ1 (jasmonic acid), whereas heat-killed conidia induced modest early increases in ERF1 and MPK2 with no significant effect on WRKY and JAZ1. Phytohormones are responsible for inducing a massive reprogramming in plant transcriptional networks that culminate in the increased production of numerous anti-herbivore traits and plants impaired the perception of these hormones are unable to mount a suitable defense response [161,162]. For example, JA-defective plants are impaired in multiple defense traits and become highly susceptible to a broad range of insects, and even to uncommon non-insect herbivores like molluscs [163,164,165,166]. 

Jasmonates (JAs) are among the key regulators of non-host resistance (NHR) and play important roles in plant defense signaling. Disruption of JA signaling can render normally resistant plants susceptible to non-adapted insects, highlighting its role in host selection [167,168,169,170,171]. In addition, JA regulates several defense mechanisms, including the production of proteinase inhibitors, toxic secondary metabolites, and VOCs. Impairment of JA signaling can reduce the production or effectiveness of these defenses [170,172]. However, NHR results from the integration of diverse defense mechanisms, including preformed barriers, pathogen recognition systems, and interconnected SA-, JA-, and ET-dependent signaling networks. Within this complex defense system, JA signaling plays a pivotal role in coordinating multiple anti-herbivore and anti-pathogen defenses, and alterations in JA biosynthesis or signaling can substantially affect plant resistance outcomes. Consequently, targeted manipulation of JA biosynthesis and signaling may contribute to improving crop resistance to pests and pathogens, with potential benefits for agricultural productivity and sustainability [173].

Several rhizosphere bacteria and fungi produce phytohormones (auxins, gibberellins, cytokinins) which promote plant growth and increase crop yields [174,175]. EEPF also produce plant hormones, notably the auxin indole-3-acetic acid (IAA), which is best known for its role in plant cell elongation, division, and differentiation. Different species of *Metarhizium* (*M. robertsii*, *M. humberi*, *M. anisopliae*, *M. marquandii*), *Purpureocillium* and *Beauveria* (e.g., *B. brongniartii*) have been shown to produce IAA and promote plant growth [27,28,176,177]. *M. robertsii* promotes lateral root growth and root hair development of *Arabidopsis* seedlings in part through an IAA-dependent mechanism [176].

Besides phytohormones, EEPF have also been reported to trigger ROS production, such as superoxide and hydrogen peroxide, to protect plants under pest attack [178]. The response also involves activation of mitogen-activated protein kinases (MAPKs), which trigger metabolic changes, extensive transcriptional reprogramming, and the production and accumulation of phytoalexins and other secondary metabolites [159]. These processes lead to localized programmed cell death, termed the hypersensitive response (HR), which helps limit the spread of pathogens [179,180] (Figure 4).

## 3. EEPF Impact on Herbivore Feeding, Development, and Survival

EEPF, along with many other plant-associated microorganisms, play important roles in defending plants against insect infestation and damage through both direct and indirect mechanisms [20,159]. They can also modify the behavior and performance of insect pests and their natural enemies [42,181,182]. EEPF elicit physiological changes in the host plant which impact plant–herbivore interactions. Most often, the physiological changes negatively impact herbivory, development, survival, and oviposition, with relatively few exceptions (Table 1, Table 2 and Table 3). However, the degree of the effect depends on the fungal strain, host plant, and pest species. It is important to note that mycoparasitic fungi like *Trichoderma* spp. and opportunistic pathogens such as *Aspergillus* spp. can also induce plant changes that affect multitrophic interactions [183,184,185,186]. However, here we focus specifically on the effects of EEPF on the pest complex, and mycoparasites will be referenced only when relevant. The following sections examine the impact of EEPF on pest herbivory, development, and survival, interconnected processes, as reduced feeding can directly influence both insect development and survival.

### 3.1. Effect of EEPF on Insects and Mites with Sucking Mouthparts

#### 3.1.1. Infection, Mortality, and Mycosis in Piercing–Sucking Arthropod Pests

EPF infect insect hosts through a sequential process of cuticular penetration, haemocoel colonization, and finally sporulation on the cadavers [4]. Insects with piercing–sucking mouthparts generally show limited evidence of ingesting fungal propagules or attachment of spores to the areas around the mouthparts, partially due to a reduced likelihood of direct spore contact and a reduced amount of articulated mouthparts, which offer the conidia access to the integument, which often serves as an entry point [187].

Interestingly, EEPF may affect piercing–sucking herbivores through indirect, plant-mediated mechanisms arising from changes in host plant chemistry and defense responses. Current evidence suggests that many of the observed effects on herbivores are associated with EEPF-induced modifications in plant chemistry, including changes in defensive metabolites and signaling pathways. However, the extent to which fungal secondary metabolites contribute to these responses remains unclear, because their occurrence and distribution within plant tissues are not yet fully understood (Section 2). Together, these changes have the potential to alter the chemical environment encountered by herbivores, including exposure to compounds with toxic or deterrent properties (Table 1; Figure 2) [31,77,188].

Numerous studies have documented significant mortality in piercing–sucking insects feeding on endophytically colonized plants (Table 1). For example, tomato plants inoculated with *Cordyceps javanica* (=*Isaria javanica*) and *Purpureocillium lilacinum* (=*Paecilomyces lilacinum)* caused 52% and 45% mortality of *B. tabaci* adults, respectively [189]. *C. fumosorosea*-colonized eggplant (*Solanum melongena*) significantly reduced *B. tabaci* incidence and pupal survival [190]. Tomato plants colonized with *B. bassiana* caused mortality in *T. vaporariorum* adults and nymphs, although susceptibility varied among developmental stages [188]. Mortality of *B. tabaci* nymphs feeding on *B. bassiana*-colonized melon reached 92% [31]. In tomato, however, the effects were less pronounced, with endophytic *B. bassiana* reducing survival and reproductive performance of *B. tabaci* [54]. Melon plants colonized with *B. bassiana* induced significant mortality in *A. gossypii*; however, within the same study, *M. anisopliae* had a weaker effect, highlighting the importance of strain selection based on the target pest and crop [191]. Similarly, the inoculation method strongly influenced the efficacy of endophytic *B. bassiana* in cabbage, with *M. persicae* mortality ranging from 62% under soil drenching to 92% under combination treatments, while intermediate mortality levels were observed with foliar spray (87%), seed treatment (67%), and root dip inoculation (76%) [192].

Mycosis mediated through piercing–sucking feeding modes appears to be rare. For example, *B. tabaci* and *M. persicae* feeding on melon and tobacco colonized by *B. bassiana* exhibited increased mortality and signs of infection [31,193]; however, clear evidence of consistent fungal outgrowth from cadavers remains limited. These cases therefore appear to represent exceptions rather than the general rule. Further investigation into the mechanisms underlying these interactions is needed to determine whether fungal metabolites, plant-mediated defenses, or occasional direct infection are responsible, and whether such processes could be exploited to enhance dissemination and persistence of EEPF in agroecosystems.

#### 3.1.2. Development, Fitness, Fecundity, and Survival in Piercing–Sucking Pests

EEPF can impair piercing–sucking insects’ feeding and physiology, reducing survival and reproduction indirectly through plant-mediated defenses and altered phytochemistry (Figure 2). Aphid longevity and fecundity declined by 18% and 10%, respectively, when feeding on sweet pepper colonized with *A. muscarius* [97]. Likewise, cotton plants colonized with either *Lecanicillium lecanii* or *B. bassiana* saw significantly reduced *A. gossypii* reproduction [194]. *Vicia faba* plants colonized by *B. bassiana* resulted in reduced reproductive potential and overall populations of both *Aphis fabae* and *Acyrthosiphon pisum*, while endophytic *M. anisopliae* had no effect, highlighting the importance of selecting for compatibility between plant and fungus [195]. Seyab et al. [196] showed that brown planthopper (*Nilaparvata lugens*) feeding on *M. anisopliae*-colonized rice plants had reduced survival, weight, honeydew secretion, and fecundity. Furthermore, female brown planthoppers preferred to feed and lay eggs on non-colonized plants.

In bean plants, endophytic *B. bassiana* negatively affected the western flower thrips, *Frankliniella occidentalis*, resulting in reduced feeding preference, prolonged preoviposition periods, decreased fecundity, and extended developmental duration [197].Similarly, *Citrus limon* plants colonized by *B. bassiana* have been shown to reduce the fecundity and performance of *Diaphorina citri* [198]. Similar reductions were seen in *Sitobion avenae* populations raised on *B. bassiana*-colonized maize [199] (Table 1).

Two of the most important commercial EPF strains, *M. brunneum* strain F52 and *B. bassiana* strain ATCC74040, have also been found to significantly reduce reproduction in *M. persicae* aphids when used as an EEPF in sweet pepper plants [80]. This study was particularly interesting as, despite the overall population decreasing, winged adult numbers increased, perhaps due to stress induction alongside decreased host plant suitability resulting in a pressure to colonize new plants.

The effects of EEPF on fecundity and reproduction are not limited to insects; several mite species (Acari) are also affected. Spider mites, particularly those from the genus *Tetranychus*, represent one of the most important agricultural pest groups across a wide range of crops, are also affected significantly. *Beauveria bassiana* colonizing bean plants as an endophyte successfully decreased red spider mite *Tetranychus urticae* fecundity and lifespan [200]. Similarly, tomato plants colonized by *M. anisopliae* and *M. brunneum* lowered *T. urticae* oviposition and population growth [201,202], showcasing the broad potential of several EPF genera across different crop plants and against a range of arthropod pest species (Table 1).

**Table 1:** Tritrophic interactions involving endophytic entomopathogenic fungi, host plants, and their effects on piercing–sucking insects, summarizing the diversity of biological outcomes including pest mortality, reduced fitness, altered behavior, plant-mediated resistance responses, and context-dependent effects that vary with fungal strain, host plant, application method, and insect species.

Fungi (Isolate/Strain)	Host Plant	Target Pest	Biological Impact	Reference
*Acremonium strictum*	*Solanum lycopersicum*	*Trialeurodes vaporariorum*	Colonization induces mortality across all life stages under drought conditions; despite high mortality, initial colonization did not deter insect preference for the plant.	[128]
*Beauveria bassiana* *Lecanicillium lecanii* *Aspergillus parasiticus*	*Gossypium hirsutum*, *Triticum aestivum*, *Phaseolus vulgaris*, *Zea mays*, *S. lycopersicum*, *Cucurbita maxima*	*Aphis gossypii*, *Chortoicetes terminifera*	Retard reproductive rates in aphids and cause significant stunting of nymphal growth in grasshoppers following ingestion of colonized tissue.	[194]
*Hypocrea lixii* (F3ST1) *B. bassiana* (GILU3) *T. asperellum* (N1LT6)	*Vicia faba*	*Acyrthosiphon pisum*, *Aphis fabae*	Significant suppression of population density; colonization caused marked delays in developmental onset and reproductive maturity.	[195]
*B. bassiana*	*S. lycopersicum*	*Bemisia tabaci*	A 90% mortality was recorded in *B. tabaci* populations.	[203]
*B. bassiana* (GHA) *Purpureocillium lilacinum*	*G. hirsutum*	*A. gossypii*	Sustained negative impact on reproductive capacity observed over a prolonged 14-day exposure period. Significantly reduced survival, fecundity, and population growth of *Aphis gossypii* over a 14-day feeding period.	[204]
Clonostachys rosea (707) *Trichoderma asperellum* (M2RT4) *H. lixii* (F3ST1)	*Allium cepa*	*Thrips tabaci*	Reduced oviposition and feeding punctures of *T. tabaci*. (mechanical damage).	[205]
*H. lixii* (F3ST1)	*A. cepa*	*Tetranychus urticae*	Reduction in oviposition; females displayed a 3.3-fold behavioral preference for control plants. Reduced oviposition of *T. urticae* and shifted female host preference toward non-colonized onion plants, indicating strong volatile-mediated deterrence.	[122]
*Phialemonium inflatum* (TAMU490) *B. bassiana* (GHA)	*G. hirsutum*	Lygus *hesperus*, *Nezara viridula*	Colonized cotton tissues deterred settling and feeding-associated behaviors of *L. hesperus* and *N. viridula*.	[206]
*B. bassiana* (Bb04, EABb 01/33-Su) *Metarhizium brunneum* (EAMb 09/01-Su)	*Cucumis melo*	*B. tabaci*	Mortality reached 92.25% of nymphs; detection of *dtxA* mycotoxins in 43% of nymphs indicates ingestion of fungal secondary metabolites.	[31]
*B. bassiana* (B12, B13, B16), *Isaria fumosorosea* (I7)	*P. vulgaris*	*T. urticae*	Impaired larval survival and exerted negative impacts on the development, fecundity, and fitness of subsequent generations.	[207]
*M. brunneum* (F52) *B. bassiana* (GHA)	Glycine max	*Aphis glycines*, *Heterodera glycines*	*Beauveria bassiana* effectively suppressed aphids, while *M. brunneum* suppressed nematode populations; notably, *M. brunneum* increased aphid size.	[208]
*B. bassiana* *M. brunneum*	*Capsicum annuum*	*Myzus persicae* *Aphidius colemani*	Feeding on colonized plants reduced the development and fecundity of first- and second-generation *M. persicae* but did not affect *A. colemani*.	[80]
*B. bassiana* (ATCC 74040, GHA)	*Vitis vinifera*	*Planococcus ficus*, *Empoasca vitis*	General suppression of infestation density.	[209]
*B. bassiana* (EABb 04/01-Tip, -33-Su) *M. brunneum* (EAMa 01/58-Su)	*C. melo*	*A. gossypii*	Accelerated initial fecundity but reduced overall nymphal output; foliar fungal suspensions significantly increased mortality.	[191]
*B. bassiana* *I. fumosorosea* *Metarhizium anisopliae*	Strawberry (*Fragaria × ananassa*)	*Myzus persicae*	Disruption of pre-feeding behavior; aphids showed fewer short probes and a high propensity to abandon colonized leaves.	[210]
*B. bassiana* (BBFafu-13, 16) *I. fumosorosea* (IFFafu-1)	Citrus limon	*Diaphorina citri*	Significant reduction in adult survival, with a 50% mortality rate within only 5 days of exposure to colonized host plants.	[211]
*B. bassiana* (GHA)	*Z. may*	*Sitobion avenae*	Inoculated maize reduced survival by 49%. Reduced nymphs by 74%	[199]
*B. bassiana* (RGM-547, 557, 570, 644, 731)	*S. lycopersicum*	*T. vaporariorum*	Significant reduction in oviposition and nymphal densities; specific strains (RGM-557, -644) outperformed synthetic insecticides.	[212]
*B. bassiana* (TV)	*S. lycopersicum*	*T. vaporariorum*	Enhanced plant defense responses characterized by elevated total phenolics and protein content, leading to high nymphal mortality.	[188]
*M. brunneum* (Met52)	*Brassica oleracea*	*Delia radicum*	Altered volatile profiles and leaf color, which unexpectedly increased attraction and oviposition by cabbage root flies.	[130]
*B. bassiana* (Bb252)	*S. lycopersicum*	*B. tabaci*	Most *B. tabaci* preferred feeding on uninoculated leaves over *B. bassiana*-treated leaves.	[213]
*B. bassiana* (GHA)	*Carya illinoinensis*	*Melanocallis caryaefoliae* *Monellia caryella*	Reduced populations of two pecan aphids when placed on colonized leaves.	[214]
*B. bassiana* (LPSC 1067)	*C. annuum*	*M. persicae*	A tendency for increased adult mortality and reduced offspring (nymphs) in insects reared on colonized plants	[215]
*B. bassiana (Bb-Taif1)*	*Vitis vinifera*	*Aphis illinoisensis*	Elevated aphid mortality was recorded in the Bb-Taif1 treatment	[216]
*B. bassiana* (GHA)	*V. faba*	*A. fabae, Aphidius colemani* *	Colonization did not affect aphid preference or parasitization rates but reduced adult emergence of *A. colemani*, coinciding with elevated expression of defense-related genes associated with induced resistance responses.	[217]
*Cordyceps fumosorosea*	*Solanum melongena*	*B. tabaci*	Reduced *B. tabaci* incidence, egg hatchability, and pupal survival.	[190]
*M. robertsii* (ESALQ 1622) *B. bassiana* (GHA)	*T. aestivum*	*Rhopalosiphum padi*, *A. fabae*	Seed-based inoculation effectively reduces population densities in cereals and beans by limiting aphid establishment.	[218]
*B. bassiana* (LTB01)	*P. vulgaris*	*T. urticae*	Activated multiple plant defense pathways. Negatively affected mite growth and performance in a stage-dependent manner. The herbivore simultaneously upregulated detoxification genes as a coping mechanism in the colonized plant tissues.	[219]
*B. bassiana* (EABb 01/33-Su)	*C. melo*	*A. gossypii*	Fungi-altered melon VOC emissions, distinct from uncolonized plants. Some VOCs released are known to influence herbivore-induced signaling, natural enemy attraction	[98]
*B. bassiana* (GHA) Metarhizium *acridum* (IMI330189)	*Nicotiana tabacum*	*M. persicae*	Increased mortality and reduced fecundity; *M. acridum* specifically delayed the transmission/infection of the PLRV virus.	[220]
*B. bassiana* *M. anisopliae*	*C. sativus*	*A. gossypii*	Significant negative effect on *A gossypii* population size after 5 days of exposure to colonized plants.	[221]
*B. bassiana*	*Nicotiana benthamiana*	*M. persicae*	Colonized seedlings displayed higher resistance to aphids.	[222]
*B. bassiana* (PPRI 5339) *M. brunneum* (Mb7)	*S. lycopersicum*	*T. urticae*, *Tuta absoluta*	Reduced populations and feeding damage of multiple tomato pests compared to control.	[155]
*B. bassiana*	*Citrus limon*	*Diaphorina citri*	Reduced fecundity, survival, and overall performance of *Diaphorina citri*.	[198]
Akanthomyces *muscarius* (5128) *B. bassiana* (3097)	*C. annuum*	*M. persicae*	Negatively affected aphid longevity and fecundity; and plants remained attractive to aphids via odor, leading to a “trap-like” effect.	[97]
*B. bassiana* (GHA, PPRI 5339, AP0101)	*C. melo*, (*Fragaria × ananassa*),	*A. gossypii*, *Frankliniella occidentalis*	Significantly reduced populations in both aphids and thrips across multiple host species via plant-mediated effects.	[223]
*B. bassiana* (UHSB-END)	*B. oleracea*	*M. persicae* *P. xylostella*	Significant mortality of *M. persicae* and *P. xylostella* was observed on cabbage treated with *B. bassiana*, depending on the inoculation method.	[192]
*Isaria javanica* *P. lilacinum*	*S. lycopersicum*	*B. tabaci*	High adult mortality and reduced adult emergence rates, with emergence rates remaining substantially higher in control treatments.	[189]
*B. bassiana*	*S. lycopersicum*	*B. tabaci*	Significantly reduced oviposition of *B. tabaci* under both root irrigation and foliar inoculation treatments relative to controls.	[54]
*B. bassiana* (GHA, GxABT-1)	*Beta vulgaris*	*M. persicae*	Altered life cycle progression and reduced viral load/transmission efficiency for plant viruses.	[224]
*M. anisopliae* (WEU01, WEU02)	*S. lycopersicum*	*T. urticae*	Reduced *T. urticae* populations, oviposition.	[202]
*Cordyceps cateniannulata*	*S. lycopersicum*	*T. urticae*	Leaf colonization rates were positively correlated with inhibition of *T. urticae* populations.	[225]
*B. bassiana* (GZGY-1-3, XJWLMQ-32)	*P. vulgaris*	*F. occidentalis*	Preference for control leaves; induced extended preoviposition periods, reduced fecundity, and slowed egg/nymph development.	[197]
*M. brunneum* (KVL16-36, KIS1868) *C. rosea* (KIS1881)	*Fragaria × ananassa* (Clery, Faith, Rumba)	*T. urticae*	Leaf applications reduced mite survival and slowed development, while some root applications unexpectedly increased mite populations, indicating that fungi effects are dependent on the application method.	[226]
*M. bunneum* (1868) *C. rosea* (1881) Arbuscular mycorrhizal fungi	*Fragaria × ananassa*	Herbivores, natural enemies (e.g., predatory mites, lacewings, hoverflies)	The effects of fungal bioinocula on strawberry arthropod communities were context-dependent, with increased predator abundance but reduced overall arthropod diversity. Organic farming systems supported greater arthropod abundance and diversity than integrated pest management systems.	[227]
*M. brunneum* (EAMa 01/58-Su)	*Olea europaea*	*Philaenus spumarius*	Significantly altered feeding behavior, increasing probing and unsuccessful xylem access compared with controls. This indicates reduced feeding efficiency, with stronger effects in foliar sprayed than systemically colonized plants.	[228]
*M. anisopliae*	*Rice*	*Nilaparvata lugens*, *BPH*	Colonization by *M. anisopliae* can negatively affect brown planthopper performance and behavior while promoting rice growth.	[196]

* Natural enemy of pest (i.e., Parasitoid, predator); ethylene (ET), jasmonic acid (JA), salicylic acid (SA), superoxide dismutase (SOD), peroxidase (POD), catalase (CAT).

#### 3.1.3. Behavior of Piercing–Sucking Arthropods on EEPF-Colonized Plants

In piercing–sucking arthropods, the ability to detect suitable feeding sites is critical for guiding behavior. These insects commonly rely on olfactory and other plant-derived chemical cues, alongside visual and environmental signals, to locate and evaluate potential host plants [226,227]. Many species exhibit pre-feeding behaviors that determine whether they remain on or leave a plant. For example, aphids perform a series of brief, exploratory stylet probes shortly after landing, allowing them to assess plant suitability and ultimately accept or reject the host [210,228].

As outlined earlier, EEPF may indirectly influence pest behavior by altering host plant cues, highlighting their potential relevance to IPM [98,229]. A better understanding of these ecological interactions may facilitate the strategic deployment of EEPF and enhance the effectiveness of EEPF-based pest management approaches [181].

There are conflicting reports of piercing–sucking arthropod responses to plants colonized by EEPF. Some studies have shown that herbivores are attracted to plants inoculated with EEPF [97,128,130], while others have shown avoidance of EEPF-colonized plants [204,206,213,223]. Among aphids, *M. persicae* was found to prefer sweet pepper plants inoculated with *B. bassiana*, *M. acridum*, or *A. muscarius* over uninoculated plants [97,220], while Manoussopoulos et al. [210] reported altered pre-feeding behavior of *M. persicae* on strawberry plants colonized by *B. bassiana*, *I. fumosorosea*, and *M. anisopliae* var. robertsii, including increased leaf abandonment and reduced probing activity, possibly due to fungus-induced deterrent effects. Responses have also been reported in spider mites; tomato plants colonized by *B. bassiana* or *Cordyceps cateniannulata* negatively affected spider mite performance and reduced plant damage caused by *Tetranychus evansi* and *T. urticae* [225,230]. Likewise, the western flower thrips, *Frankliniella occidentalis*, were also repelled by *B. bassiana*-colonized tomato plants [197]. The brown planthopper also showed avoidance of *M. anisopliae*-colonized rice [196] (Table 1). These behavioral responses are assumed to be related to the alteration of VOC emissions outputs from the plants, and, in the case of repellency, are likely tied to arthropod recognition of increased repellent plant metabolites or endophyte-induced signals [231,232,233]. However, these contrasting outcomes highlight the need for a full understanding of the plant–fungus–pest interactions.

Interestingly, some endophytic mycoparasitic fungi have also been shown to influence insect behavior and reduce pest populations. For example, *Trichoderma asperellum* significantly reduced *Acyrthosiphon pisum* abundance, whereas *H. lixii* had no detectable effect [195]. Similarly, mycoparasitic fungi can alter plant attractiveness to herbivores; onion plants colonized by *Clonostachys rosea*, *T. asperellum*, *T. atroviride*, or *H. lixii* exhibited significantly fewer feeding and oviposition punctures compared with control plants under field conditions [205]. These findings indicate that endophytic mycoparasitic fungi can modulate pest behavior and population dynamics, although their effects appear to be species- and strain-dependent. This variability highlights the need for a more mechanistic understanding of mycoparasitic fungi –plant–insect interactions. Such knowledge could inform IPM strategies, particularly where mycoparasitic fungi and EPF are co-deployed to simultaneously suppress plant pathogens and insect pests [234,235].

#### 3.1.4. EEPF-Mediated Modulation of Virus–Vector Interactions

Piercing–sucking arthropods such as aphids, whiteflies, and mealybugs transmit viruses that cause more than US $30 billion in annual crop losses [236]. By impairing vector performance and altering insect behavior, EEPF may contribute to IPM strategies aimed at reducing the spread of insect-vectored plant viruses.

Evidence suggests that EEPF can lower virus incidence [220], although the mechanisms remain unclear. Effects may be plant-mediated through changes in volatile emissions and defense metabolites that alter vector feeding, movement, and virus acquisition or may involve interference with virus replication in plant tissues [181,191,237].

Several studies support plant-mediated effects. For example, *B. bassiana* reduced zucchini yellow mosaic virus in squash (*Cucurbita pepo* L.) more than endophyte-free plants [238]. Similarly, *M. anisopliae* decreased sugarcane mosaic virus severity in maize, but no significant effect was detected for maize chlorotic mottle virus, suggesting virus-specific responses [239]. *Lecanicillium lecanii* lowered bean common mosaic virus in yard-long bean (*Vigna unguiculata* subsp. *sesquipedalis*), potentially due to antifungal-associated antiviral factors [240]. Similarly, *Neotyphodium uncinatum* colonized meadow ryegrass (*Lolium pratense*) reduced bird cherry oat aphid *Rhopalosiphum padi* numbers and barley yellow dwarf virus infection, indicating that endophyte colonization may protect meadow ryegrass from BYDV [241].

Beyond direct effects on virus accumulation, EEPF may alter host plant metabolism in ways that enhance tolerance to viral infection. Endophytic colonization by *B. bassiana* and *M. anisopliae* has been shown to induce extensive metabolic reprogramming, including modulation of amino acid, organic acid, and phenylpropanoid pathways, which are closely linked to plant defense responses [221]. Although these biochemical changes do not necessarily reduce viral titers, they may be associated with reduced symptom severity or improved plant performance under viral infection in some experimental systems.

EEPF also influence virus epidemiology by directly affecting vector biology and transmission efficiency. Colonization by *B. bassiana* significantly reduced survival, fecundity, and transmission efficiency of *M. persicae* carrying potato leafroll virus, thereby delaying virus spread [220]. Furthermore, viruliferous vectors feeding on EEPF-colonized plants often exhibit increased physiological stress and reduced performance [181]. These observations suggest that EEPF-induced changes in host plants may contribute to reduced vector fitness and transmission potential, although the underlying mechanisms remain incompletely understood.

Overall, EEPF may suppress plant virus epidemics through multiple interacting mechanisms: reducing vector populations, altering vector behavior, enhancing plant defenses, and possibly limiting viral replication. While mechanisms require further study, EEPF integration into IPM offers a sustainable strategy for managing insect-borne plant diseases.

### 3.2. Endophytic EPF Effect on Insects with Chewing Mouthparts

#### 3.2.1. Infection, Mortality, and Mycosis in Chewing Herbivorous Pests

In contrast to piercing–sucking insects, chewing herbivores can acquire EPF infections not only via cuticular contact but also through ingestion of fungal propagules or spore adherence to mouthparts, leading to infection and mortality [3]. Notably, indirect exposure through feeding on plants colonized by EEPF often results in considerable mortality, although visible mycosis is infrequent. This pattern suggests that plant-mediated effects, rather than direct fungal infection, are the primary drivers of insect suppression.

Empirical evidence supports this interpretation. For example, *Plutella xylostella* larvae exhibited >70–80% mortality when fed cabbage previously inoculated with *M. anisopliae* or *B. bassiana,* particularly following foliar inoculation [242]. Similarly, endophytic colonization of *Brassica napus* by *M. anisopliae* resulted in 63.3% mortality of *P. xylostella* four weeks after inoculation, suggesting prolonged EEPF activity [243]. Moderate but significant increases in *S. littoralis* mortality have also been observed on EEPF-colonized melon [244], suggesting that these effects extend across multiple chewing insect species. However, responses are not universal; no mortality was detected in *S. littoralis* feeding on *B. vulgaris* colonized by *B. bassiana* or *B. varroae* [51], underscoring the need for system-specific validation prior to implementation in IPM programs (Table 2).

Consistent with patterns observed in piercing–sucking insects, mycosis in externally feeding chewing herbivores is generally rare. Seed inoculation of maize with *B. bassiana* GHA induced mortality in *Helicoverpa zea*, yet only 4.6% of individuals exhibited mycosis [245]. Likewise, low mycosis rates were observed in *P. xylostella* (2%) and *Myzus persicae* (<5%) feeding on fungus-colonized cabbage plants [192], as well as in *Spodoptera litura* feeding on colonized tomato plants (1%) [246]. In other cases, *B. bassiana* strains ANT-03 and GHA caused 8% and 35% mortality in *H. zea*, respectively, without any observable mycosis [247]. These observations suggest that plant-mediated mechanisms may contribute to the effects of EEPF on herbivores, although the potential contribution of direct fungal interactions cannot be excluded.

Exceptions do occur, and mycosis has occasionally been reported in insects feeding on EEPF-colonized plants [214,245]. For example, systemic colonization of banana by *B. bassiana* resulted in 53.4–57.7% mortality and 23.5–88.9% mycosis in *Cosmopolites sordidus* [248]. Such cases are most commonly observed in chewing herbivores that may become directly exposed to fungal hyphae emerging from wounded plant tissues during feeding. Under these circumstances, epiphytic conidiation and subsequent infection can occur, resulting in visible mycosis on insect cadavers. Powell et al. [245] suggested that infection may arise when insects consume sufficient quantities of intact fungal hyphae. This variation underscores the importance of expanding investigations beyond model pest systems to include a broader diversity of insect taxa, to better understand the ecological significance and mechanisms of EEPF-mediated insect suppression (Table 2).

EEPF-associated mycosis is more frequently reported in insects with rasping–chewing feeding modes, such as leaf miners. In horse chestnut, *B. bassiana* colonization caused 34.66–56.81% mortality in *Cameraria ohridella*, with 5.41–9.23% mycosis observed in larvae and pupae [249]. In *T. absoluta*, foliar applications of *B. bassiana* achieved 90–100% mortality, whereas endophytic colonization of tomato resulted in 33–52% larval mortality alongside 30–50% mycosis, indicating both external and internal modes of action [250] (Table 3). Nevertheless, high mortality in the absence of mycosis has also been widely documented in rasping–chewing taxa. For example, *T. absoluta* and *Ostrinia furnacalis* feeding on EEPF-colonized plants exhibited elevated mortality without fungal outgrowth from cadavers [37,125]. Similarly, *Liriomyza huidobrensis* mortality on colonized *V. faba* occurred without visible mycosis [37]. Comparable outcomes have been reported following topical EPF applications, suggesting that fungal-induced mortality does not necessarily culminate in external sporulation [251,252].

Collectively, these findings indicate that EEPF-mediated suppression of herbivores is driven by multiple interacting mechanisms, including plant-induced defense responses, exposure to fungal- and plant-derived metabolites, and sublethal physiological disruption. This is consistent with the endophytic lifestyle of EEPF, in which the fungus grows as vegetative mycelia within plant tissues without producing infective structures, unlike its development within the hemolymph of insect hosts. Consequently, herbivores feeding on colonized plants generally do not develop mycosis, and the absence of visible fungal outgrowth should not be interpreted as evidence of failed infection or fungal inactivity, as fungal development and metabolite production may occur internally without external sporulation [253].

**Table 2:** Tritrophic interactions involving endophytic entomopathogenic fungi, host plants, and their effects on chewing insects, summarizing the diversity of biological outcomes, including pest mortality, reduced fitness, altered behavior, plant-mediated resistance responses, and context-dependent effects that vary with fungal strain, host plant, application method, and insect species.

Fungi (Isolate/Strain)	Host Plant	Target Pest	Biological Impact	Reference
*Acremonium strictum*	*S. lycopersicum*	*Helicoverpa armigera*	Reduced growth rates and prolonged development; smaller pupae with high mortality; reduced adult fecundity.	[129]
*M. anisopliae* (F52)	*Z. mays*	*Agriotes obscurus*	Recovery of cadavers with fungal growth; reduced wireworm damage and increased maize yield.	[254]
*A. strictum*	*S. lycopersicum*	*H. armigera*	Altered VOC profiles influenced and deterred moth oviposition behavior.	[100]
*B. bassiana* (11–98)	*S. lycopersicum*	*H. zea* (3rd instar)	Mycosis reported in 4.6% of the larvae fed on colonized plants.	[245]
*A. strictum*	*V. faba*	*H. armigera*	Reduced survival and prolonged prepupal/pupal stages; significant reduction in 2nd-gen fecundity.	[255]
*B. bassiana* (ITCC 6063, ITCC 4512, ITCC 4563, ITCC 5562, ITCC 4796, ITCC 5408)	*Corchorus capsularis*	*Apion corchori*	Reduced stem weevil infestation under pot culture.	[256]
*B. bassiana* (Bb03032, ATP01, ATP02)	*V. faba*	*H. armigera*	Significantly higher larval mortality; mycosis confirmed in affected larvae.	[72]
*Metarhizium pingshaense* (MGC02, MGC06)	*Z. mays*	*Agriotes cinctus*	A 50% infection rate via seed treatment; 93% via direct soil inoculation.	[257]
*B. bassiana* (WG-40)	*S. lycopersicum*	*H. armigera*	Significantly increased mortality of larvae and reduced pupation and adult emergence rates.	[253]
*B. bassiana* *P. lilacinum*	*G. hirsutum*	*H. zea*	Insects feeding on endophyte-colonized plants exhibited reduced survival rate	[258]
*B. bassiana*	*S. lycopersicum*	*Spodoptera exigua*	Reduced *S. exigua* larval weight	[49]
*B. bassiana* (BPRC-F1, -F2)	*Pinus radiata*	*H. armigera, Costelytra zealandica*	Reduced survival of *H. armigera* larvae by more than 10% and decreased larval weight of *C. zealandica* by approximately 5%.	[139]
*B. bassiana* *M. brunneum*	*C. melo, Medicago sativa, S. lycopersicum*	*Spodoptera littoralis*	Mortality of *larvae* ranged from 25–46.7% following endophytic fungal colonization and increased to ~80% when combined with conidial applications.	[259]
*B. bassiana*	*Z. mays*	*Spodoptera frugiperda*	Seventy-five percent of larvae feeding on *B. bassiana*-inoculated plants died by day 14.	[260]
*B. bassiana* (B2)	*B. oleracea*	*Plutella xylostella*	Marked reduction of larval survival without visible mycosis; no effect on oviposition preference.	[261]
*B. bassiana* (LPSC 1067)	*Z. mays*	*Dichroplus maculipennis*	Colonization of maize suppressed feeding, oviposition, and egg embryonation of *D. maculipennis.*	[262]
*B. bassiana* Mycorrhizal fungi	*Z. mays*	*Phyllophaga vetula*	Combined inoculation with AMF and *B. bassiana* enhanced maize performance, although only ~5% of *P. vetula* larvae became infected by the fungus.	[263]
*B. bassiana* (B2) *Bacillus subtilis*	*S. lycopersicum*	*H. armigera*	Stimulated defense-related enzyme activity in tomato plants, contributing to increased resistance against *H. armigera*.	[264]
*B. bassiana* (LPSC 1215)	*N. tabacum*	*H. gelotopoeon*	Reduced larval development and fecundity, shortened the oviposition period, and did not deter feeding.	[265]
*B. bassiana* EABb 04/01-Tip	*Triticum aestivum* *Triticum durum*	*S. littoralis* (larvae)	Mortality of *S. littoralis* larvae fed on leaves from inoculated plants ranged from 30% to 57%, depending on the application method.	[184]
*B. bassiana* (LPSC 1098)	*Z. mays*, G. max	*Rachiplusia nu*, *H. gelotopoeon*	Reduced leaf consumption and insect feeding preference; decreased fecundity and fertility.	[140,266]
*M. brunneum* (EAMb 09/01-Su)	*Z. mays*	*S. littoralis*	Reduced leaf consumption, lower fitness, and decreased egg fecundity/fertility.	[244]
*B. bassiana* (Bb-18) *M. anisopliae* (Ma-30)	*Z. mays*	*Spodoptera frugiperda*	100% mortality in 2nd instars; 75–87% mortality in 4th instars feeding on treated plants.	[267]
*Metarhizium* spp. (ESALQ 1638, 1814, 2450)	*Z. mays*	*S. frugiperda*	Fungi approximately doubled the mortality rate of larvae feeding on corn, leading to significantly lower survival after 7 days compared to the control group.	[268]
*M. robertsii*	*Z. mays*	*Agrotis ipsilon*	Suppressed growth rate of black cutworm (*A. ipsilon*) larvae.	[53]
*B. bassiana* (NBAIR, Bb-5a, NBAIR Bb-45) *M. anisopliae* (NBAIR Ma-4, NBAIR Ma-35)	*B. oleracea*	*P. xylostella*	All four isolates caused varying mortality levels depending on the duration after treatment and the inoculation method.	[242]
*B. bassiana* (Bb 115)	*S. lycopersicum*	*H. armigera*	Significant reduction in larval survival and leaf consumption across multiple tomato varieties.	[269]
*B. bassiana* (BbC1) *M. anisopliae* (M150)	*S. lycopersicum* (Matina and Harzfeuer varieties)	*S. frugiperda* (2nd instar)	Reduced larval weights and slower larval development. Larval weight gain varied depending on the treated plant variety.	[270]
*M. robertsii*	*Z. mays*	*S. frugiperda*	No impact on the relative growth rate of larvae feeding on inoculated vs uninoculated maize leaves.	[271]
*B. bassiana* (EABb 01/33-Su)	*G. hirsutum*	*S. frugiperda*, *S. littoralis*	Fungi-altered cotton VOC emissions, distinct from uncolonized plants. Some VOCs released are known to influence herbivore-induced signaling and natural enemy attraction.	[98]
*M. brunneum* (EAMa 01/58-Su)	*C. melo*	*S. littoralis*, *Hyposoter didymator*	No increase in overall *S. littoralis* mortality beyond that caused by its parasitoid, although parasitoids showed a strong preference for control plants and both biological control agents were able to coexist and develop within the same host larvae.	[272]
*B. bassiana* (LPSc 1060, LPSc 1061, LPSc 1062, LPSc 1063, LPSc 1066, LPSc 1067, LPSc 1080, LPSc 1082, LPSc 1083, LPSc 1086, LPSc 1098, LPSc 1156) *M. anisopliae* (LPSc 907) *M. robertsii* (Lpsc 963)	*Z. mays*	*S. frugiperda*	Significantly reduced larval and pupal survival, prolonged developmental stages, shortened total *S. frugiperda* lifespan, and decreased leaf area consumed by third-instar larvae. Female longevity, fecundity, and fertility were also significantly reduced by endophytic *B. bassiana*.	[229]
*B. bassiana* *M. anisopliae*	*Z. mays*	*S. frugiperda*	Increased mortality on fungi-colonized plants across all larval stages, with the highest mortality (≤51%). Prolonging larval lifespan and significantly reducing pupation, adult emergence, fecundity, and egg hatch compared to non-colonized maize.	[273]
*B. bassiana* *Chaetomium* sp. *Curvularia lunata* *Penicillium citrinum* *M. anisopliae*.	*Z. mays*	*S. frugiperda*	Colonization of maize suppressed growth and development of *S. frugiperda*, with *B. bassiana* isolates JgSPK and JaGiP inducing the highest levels of pupal non-emergence.	[274]
*B. bassiana* *M. anisopliae*	*Z. mays*	*S. frugiperda*	Colonization of maize negatively affected life-table parameters of *S. frugiperda*, reducing survival, development, fecundity, and population growth rates relative to controls.	[40]
*M. brunneum* (EAMa 01/58-Su	*C. melo* *C. sativus*	*S. littoralis*	Activated plant defense networks (i.e., ET, JA, SA, and PR genes, including strong upregulation of LOX1, EIN2, and EIN3). Reduced insect survival and fitness (↑ larval mortality, prolonged development, and ↑ pupal abnormalities) compared with controls.	[144]
*B. varroae* *B. bassiana*	*Beta vulgaris*	*S. littoralis*	Pests preferred untreated plants; colonization shortened emergence periods and shifted sex ratios.	[51]
*B. bassiana* (BbofDH1-5)	*Z. mays*	*Ostrinia furnacalis*	Reduced damage from the insect and improved plant biomass and yield, with effects being stronger under elevated CO_2_; fungus also helped maintain leaf nitrogen content at levels comparable to ambient CO_2_ conditions.	[275]
*M. anisopliae* (IBCB 425)	*Cynodon dactylon*	*S. frugiperda*	Reduced fertility-related life-table parameters; slight trend toward preference in no-choice tests.	[276]
*B. bassiana* (Bb20091317) *Metarhizium rileyi*	*Z. mays*	*S. frugiperda*	Reduced larval weight; modulated benzoxazinoid genes; ZmWRKY36 regulated plant immunity.	[277]
*M. brunneum* (EAMa 01/58-Su) *B. bassiana* (EABb 04/01Tip, EABb 01/33-Su)	*C. melo*	*S. littoralis* *Hyposoter didymator*	Significantly higher larval mortality varied with the inoculation method. Larval development time increased, while larval and pupal weights were reduced. The parasitoid was compatible with all fungal strains and application methods.	[278]
*B. bassiana* (CBM1, CBM2, CBM3)	*T. aestivum*	*H. armigera*, *S. frugiperda*, *M. separata*, & *P. xylostella*.	High, rapid mortality across multiple pest species; CBM1 exhibited the highest potency.	[279]
*B. bassiana* (ChBb, NaBb)	*Z. mays*	*S. frugiperda*	Endophytic colonization caused ~51–56% mortality (compared to ~76% via direct application).	[280]
*B. bassiana* (GHA, ANT-03)	*G. hirsutum*	*H. zea*	Endophytic *B. bassiana* and neonicotinoid treatments reduced survival and feeding damage of *H. zea*, while larvae showed a preference for untreated cotton plants.	[247]
*Fusarium citri* (FcS1GZL-1)	Soybean	*S. litura*	Endophytic fungus repelled insects from feeding and enhanced soybean resistance to pathogens by upregulating 12 defense-related genes linked to jasmonic acid, salicylic acid, ethylene, and pathogen-response pathways.	[281]
*B. bassiana* (Bb11, Bb115, DL1.1)	*Zea mays*	*S. frugiperda*	Endophytic *B. bassiana* significantly reduced armyworm fitness by increasing larval mortality and suppressing growth, pupation, adult emergence, and reproduction.	[282]
*B. bassiana* (BbofDH1-5)	*Z. mays*	*O. furnacalis*	Endophytic colonization, particularly via blastospores, reduced larval feeding, growth, and survival, suppressed antioxidant enzyme activity (SOD, POD, CAT), and increased susceptibility to chlorantraniliprole through downregulation of detoxification genes and upregulation of stress-response genes	[283]

Ethylene (ET), jasmonic acid (JA), salicylic acid (SA), superoxide dismutase (SOD), peroxidase (POD), catalase (CAT).

#### 3.2.2. Development, Fitness, Fecundity, and Survival in Chewing Mouthparts Pests

EEPF affect not only mortality but also key life-history traits of chewing pests, often resulting in reduced development, fecundity, and overall fitness (Table 2). For example, *S. littoralis* exhibited reduced fecundity and egg viability when fed melon leaves colonized by *M. brunneum*, despite no detectable delay in development [244]. In the closely related *Spodoptera frugiperda*, larvae feeding on maize colonized by *B. bassiana*, *M. anisopliae*, and *M. robertsii* showed reduced larval weight, delayed development, abnormal pupation, and decreased fecundity [40,274,284]. Notably, *B. bassiana* exposure also produced a female-biased sex ratio relative to controls [285]. However, this response appears to be context-dependent, and its biological significance and underlying mechanisms remain to be established.

Comparable sublethal effects have been reported in other systems. *S. littoralis* feeding on beet leaves colonized by *B. bassiana* or *B. varroae* displayed reduced larval and pupal mass, decreased oviposition, and shifts in sex ratio from female-biased to balanced [51]. Likewise, *Helicoverpa gelotopoeon* feeding on soybean colonized by *B. bassiana* exhibited prolonged larval development and reduced fecundity and fertility, although sex ratios remained unchanged [140]. Endophytic colonization of *Salix matsudana* by *B. bassiana* altered the feeding preference of *Plagiodera versicolora*, inhibited larval weight gain and pupation, and increased mortality through plant-mediated defense response [47] (Table 2).

The mechanism behind the shift in sex ratio remains unclear. However, further studies may clarify the underlying processes and support improved strategies targeting surviving adult females to enhance post-exposure control. Even modest alterations in life-history traits, especially sex ratio and reproductive output, may substantially influence population dynamics under certain conditions. These effects may have implications for mating structure and population growth, although such outcomes have not yet been evaluated in EEPF systems. Future incorporation of these sublethal effects into population models may improve our understanding of the potential long-term consequences of EEPF for pest population dynamics and management.

Host plant genotype is a critical determinant of EEPF-mediated multitrophic interactions, often modulating both the magnitude and direction of pest suppression. For instance, *S. frugiperda* larvae feeding on the tomato cultivar ‘Matina’ colonized by *B. bassiana* or *M. anisopliae* exhibited significantly lower weights than those feeding on the inoculated ‘Harzfeuer’ cultivar [270]. This suggests that cultivar-specific traits such as constitutive or inducible defenses interact synergistically with EEPF, amplifying dietary stress and constraining larval performance, usually reflected in slow development and reduced weight.

Comparable variability is observed among rasping–chewing pests, although EEPF generally impose consistent constraints on development and reproduction. In coffee, *M. robertsii* and *M. brunneum* delayed development and reduced oviposition of the coffee leafminer, *Leucoptera coffeella* [286,287]. In beans, *M. anisopliae* and *H. lixii* reduced oviposition, pupation, and adult emergence of *Ophiomyia* spp. and *Liriomyza* spp. [37,288,289], while similar reductions in fecundity and development have been reported for *T. absoluta* on EPF-colonized tomato [124,251] (Table 3). Notably, even non-systemic fungal isolates can impair pest fitness, implying that transient colonization or induced systemic resistance may be sufficient to generate biological effects. Application method further modulates outcomes; for example, root inoculation with *B. bassiana* reduced *Liriomyza sativae* pupal densities more effectively than foliar treatments [290]. In EEPF-colonized maize, reduced survival of *Ostrinia furnacalis* and *Sesamia calamistis* suggests additional physiological or behavioral disruption [39,85]. Similarly, *P. vulgaris* colonized by *B. bassiana* exerted larvicidal effects on *Liriomyza huidobrensis* and altered sex ratios, reducing female bias [37,113].

Collectively, these findings highlight that EEPF effects are highly context-dependent, shaped by plant genotype, fungal strain, and application strategy. Crucially, sublethal effects, particularly those affecting development, reproduction, and sex ratio may have implications for pest population dynamics, although their long-term consequences remain largely unexplored in EEPF systems. Despite this, the mechanistic basis of these effects remains poorly resolved. Further research is needed to improve our understanding of these interactions and support the effective integration of EEPF into IPM programs.

#### 3.2.3. Chewing Insect Behavior in Response to Plants Colonized by EEPF

Feeding deterrence is a recurrent response of chewing herbivores to plants colonized by EEPF and is frequently attributed to ISR and shifts in plant secondary metabolites, including VOCs (Table 2). Reduced foliar consumption has been documented for *H. armigera*, *H. gelotopoeon*, and *Rachiplusia nu* on EEPF-colonized hosts [140,266,291]. However, responses are not uniform: *H. gelotopoeon* showed no reduction in feeding on *B. bassiana*-colonized tobacco despite clear negative effects on development and fertility [265]. Consistent with a deterrence mechanism, *B. bassiana* colonization also reduces incidence and survival of *H. zea* and limits *H. armigera* damage on tomato, likely via activation of plant defenses that render tissues less palatable or repellent [245,253,258,292].

Beyond direct feeding effects, EEPF can simultaneously suppress pest pressure and improve crop performance. For example, *M. anisopliae* and *H. lixii* reduced infestations of *Ophiomyia* and *Liriomyza* spp. while increasing yields [288,289]. Disentangling pest suppression from yield enhancement remains challenging under field conditions, but accumulating evidence implicates EEPF-induced changes in plant chemistry, particularly VOC emissions, as key drivers of herbivore deterrence and performance declines. Controlled assays will be essential to resolve these mechanisms.

EEPF-mediated changes also extend to oviposition behavior and higher trophic interactions, often in a pest-specific manner. In cotton, *Lygus lineolaris* and *L. hesperus* avoided *B. bassiana*-colonized tissues [293], and in sugarcane, oviposition by *D. saccharalis* declined in response to EEPF-associated changes in plant volatiles [102]. Emerging evidence suggests that EEPF may also disrupt visual host cues; *M. brunneum* alters plant spectral reflectance, reducing host recognition by *D. radicum* [130]. Although evidence remains limited, these findings suggest that EEPF-induced changes in plant traits may influence insect behavior through multiple sensory modalities. Despite these advances, behavioral responses of chewing pests to EEPF remain under-characterized, particularly relative to piercing–sucking taxa. Given the consistency of deterrence, oviposition shifts, and tritrophic effects observed to date, EEPF likely exert substantial ecological influence on chewing herbivores. Systematic investigation of these mechanisms will be critical to optimize their integration into IPM and to exploit their full potential as multifunctional BCAs.

**Table 3:** Tritrophic interactions involving endophytic entomopathogenic fungi, host plants, and their effects on rasping–chewing, summarizing the diversity of biological outcomes including pest mortality, reduced fitness, altered behavior, plant-mediated resistance responses, and context-dependent effects that vary with fungal strain, host plant, application method, and insect species.

Fungi (Isolate/Strain)	Host Plant	Target Pest	Biological Impact	Reference
*B. bassiana (ARSEF 3113)*	*Z. mays*	*Ostrinia nubilalis*	Season-long suppression; significantly reduced stalk tunnelling and damage.	[83]
*B. bassiana (ARSEF 5644-52)*	*Z. mays*	*Sesamia calamistis*	Reduced frequency of dead plants and stem-borer tunnel length.	[85]
*B. bassiana (G41)*	*Musa* sp.	*Cosmopolites sordidus*	Significant reduction in larval survivorship and adult populations; mycosis confirmed. Reduced damage to plant.	[248]
*B. bassiana* (ITCC4688)	*Sorghum bicolor*	*Chilo partellus*	Higher stem tunnelling in controls as compared to fungi-treated plants.	[294]
*B. bassiana (EABb 04/01-Tip)*	*Papaver somniferum*	*Iraella luteipes*	Reduced frequency of larvae within inoculated opium poppy stalks.	[137]
*B. bassiana* *H. lixii* *G. moniliformis* *F. oxysporum* *T. asperellum* *M. anisopliae*	*V. faba*, *P. vulgaris*	*Liriomyza huidobrensis*	A 100% larval mortality on fungi-colonized plants; *H. lixii* reduced progeny longevity and adult emergence.	[37]
*B. bassiana (B2*, *B4)*	*Arachis hypogaea*	*Aproaerema modicella*	Bioformulations increased enzyme activity; reduced leafminer damage to 2.5%.	[295]
*B. bassiana* *M. robertsii* *I. fumosorosea*	*S. bicolor*	*Sesamia nonagrioides*	Prevented 50–70% stalk entry; 70–100% larval mortality; tunnels reduced by up to 87%.	[296]
*H. lixii (F3ST1)* *B. bassiana (G1LU3)*	*P. vulgaris*	*Liriomyza leafminer* (flies)	Endophyte treatments reduced leafminer infestation, with *H. lixii* showing the greatest efficacy by producing the fewest pupae in the first season.	[289]
*B. bassiana (G1LU3*, *CIX63)* *M. anisopliae (DL7C)*	*P. vulgaris*	*Ophiomyia phaseoli*	Reduced the feeding and oviposition capacity of the female flies, and led to significantly reduced pupal weights and lower adult emergence.	[288]
*B. bassiana (ATCC 74040)*	*S. lycopersicum*	*Tuta absoluta*	The fungus caused 90–100% larval mortality through surface conidia (epiphytic action), while internal endophytic colonization reduced larval longevity and achieved 30–50% corrected mortality.	[250]
*B. bassiana (LPSC 1067)*	*S. lycopersicum*	*T. absoluta*	Reduced leaf consumption and significantly increased mortality in larvae via ingestion of endophytically colonized leaf tissues.	[297]
*B. bassiana (AM_EF0111* *AM_EP0715)* *Beauveria pseudobassiana (AM_SO1015)*	*A. hippocastanum*	*Cameraria ohridella*	Endophytic colonization by strain AM_EP0715 resulted in the leaf damage area being 5-times smaller (~80% reduction) than controls; pupae showed significantly lower weight and dimensions.	[249]
*T. asperellum (M2RT4)* *B. bassiana (ICIPE 706, ICIPE 35)* *Trichoderma* sp. *(F2L41)* *H. lixii (F3ST1)*	*S. lycopersicum*	*T. absoluta*	M2RT4, ICIPE 706, and F3ST1 significantly reduced egg-laying, mines, and pupation; F2L41 and ICIPE 35 isolates significantly lowered exposed adult and F1 progeny survival.	[298]
*B. bassiana (LEF139-LF141)*	*S. lycopersicum*	*T. absoluta*	Endophytic establishment significantly reduced larval survival (43.4% to 63.8% mortality) and extended the duration of the larval stage by several days.	[125]
*B. bassiana (OFDH1-5)* *T. asperellum* (*GDFS1009)*	*Z. mays*	*O. furnacalis*	Binary combination seed coating increased larval mortality to 90% and reduced plant stem-tunnelling metrics by over 80% via induced plant defense enzyme upregulation.	[234]
*Clonostachys* spp. (g133) *B. bassiana* (sn182)	*S. lycopersicum*	*T. absoluta*	High larvicidal efficacy at 4 × 10^7^ spores/mL with larvae mummified after 15 days; significantly reduced mined leaves in greenhouse trials.	[252]
*M. robertsii (RD-20.114)* *M. brunneum (RD-20.120)*	*Coffee*	*Leucoptera coffeella*	Root/seed inoculation reduced mined leaf area by 70%, significantly delayed larval development time, and caused emerging adults to lay significantly fewer eggs.	[286,287]
*M. anisopliae (ICIPE 20)*	*S. lycopersicum*	*T. absoluta*	Colonization caused additive effect with parasitoid *D. gelechiidivoris*; reached 80% pest mortality.	[299]
*M. flavoviride* *B. bassiana* *C. fumosorosea* *M. rileyi* *M. anisopliae*	*S. lycopersicum*	*Phthorimaea absoluta*	Endophytic colonization significantly suppressed the pest’s demographic fitness, resulting in a significantly lower net reproductive rate (R_0_) and finite rate of increase (λ).	[251]
*B. bassiana (BbHOSD1)*	*Z. mays*	*O. furnacalis*	Inoculated maize acts as a detrimental attraction; altered plant volatiles draw increased female egg-laying, but severely lower subsequent larval survival and offspring fitness.	[39]
*M. anisopliae* *B. bassiana*	*S. lycopersicum,*	*T. absoluta*	Endophytic colonization reduced pupal weight and impacted developmental time.	[300]
*M. robertsii (ESALQ 1635)*	*S. lycopersicum,* *S. pimpinelifolium,* *S. habrochaites*	*T. absoluta*	Inoculation significantly reduced oviposition by *T. absoluta* on *S. lycopersicum* and *S. habrochaites*, altered VOC profiles, and enhanced attraction of the predatory mirid bug *Macrolophus basicornis*.	[301]
*M. robertsii (ESALQ 1635)*	*Saccharum* spp.	*Diatraea saccharalis, Cotesia flavipes* *	Inoculation modulates JA/SA pathways, suppressing *D. saccharalis* oviposition in healthy plants and altering herbivore-induced volatiles to significantly attract the parasitoid wasp under pest infestation.	[102]
*Phialemonium inflatum (TAMU-490)* *B. bassiana (GHA*, *JG-1*, *NI-8*, *SPE-120)*	*G. hirsutum*	*Lygus lineolaris*	Strains JG-1 and NI-8 caused high adult mortality; JG-1 significantly modified adult olfactory behavior (repellency), while SPE-120 and NI-8 enhanced plant fruit structures (squares).	[293]
*B. bassiana (BS195*, *BNE20)*	*Cucumis sativus*	*Liriomyza sativae*	Root soaking with strain BS195 maximized colonization, significantly reducing infestation severity, total pupae counts, and adult fly emergence while enhancing plant height and total phenolics.	[290]
*B. bassiana (GHA)* *M. anisopliae (F01)*	*S. lycopersicum*	*T. absoluta*	Both endophytes induced immune-priming defense genes and secondary metabolites, reducing pest oviposition and degrading larval performance; *B. bassiana* GHA also significantly enhanced overall plant biomass.	[126]
*M. robertsii (ESALQ 1635)*	*S. lycopersicum, S. pimpinellifolium, S. habrochaites*	*T. absoluta, Macrolophus basicornis* *	Root inoculation enhanced plant biomass and altered VOC profiles, leading to a significant decrease in pest oviposition while maximizing the recruitment/attraction of the predatory bug.	[124]
*B. bassiana* *Cordyceps cateniobliqua* *Cordyceps cateniannulata* *C. fumosorosea* *M. flavoviride* *M. anisopliae*	*S. lycopersicum*	*P. absoluta, Trichogramma chilonis*	Root endophytes altered VOCs (e.g., β-phellandreneβ-phellandrene, α-phellandrene) via increased defense-related and phytohormones signaling (JA, SA, JA-Ile), enhancing tritrophic plant–herbivore–parasitoid interactions.	[96]
*B. bassiana (Bb1Bm, Bb2Bm, BbM, BbC)*	*Tomato*	*T. absoluta*	Lowered adulthood probability (36% vs 62% control); suppressed net reproductive rate (R_0_), intrinsic rate of increase (r), inite rate of increase (λ); and prolonged generation time (T).	[302]

* Natural enemy of pest (i.e., Parasitoid, predator); ethylene (ET), jasmonic acid (JA), salicylic acid (SA), superoxide dismutase (SOD), peroxidase (POD), catalase (CAT).

## 4. EEPF Influence on Natural Enemies and Multitrophic Interactions

### 4.1. EEPF-Mediated Behavior in Natural Enemies of Pest Insects

EEPF influence multitrophic interactions by altering plant traits, pest performance, and the behavior and performance of natural enemies. Although numerous studies demonstrate their potential for IPM, natural enemy responses vary in both magnitude and direction depending on the plant–fungus–arthropod system.

Natural enemies rely heavily on HIPVs for host and prey location, and EEPF colonization can modulate these cues (Section 2.3.2). Across systems, EEPF frequently alter natural enemy behavior through subtle changes in plant volatile profiles rather than large shifts in overall emissions. For example, in sugarcane, *M. robertsii* reduced attraction of *D. saccharalis* by lowering volatile emissions, while the parasitoid *Cotesia flavipes* remained responsive. When plants were both inoculated and infested, volatile emissions increased, and parasitoid attraction was enhanced, likely due to compounds such as linalool, β-elemene, and α-pinene [102]. Similarly, in sweet pepper, root inoculation with *B. bassiana* or *T. harzianum* differentially affected parasitoid behavior; *T. harzianum* enhanced attraction of *Trissolcus basalis*, whereas *B. bassiana* reduced it, despite minimal changes in overall volatile profiles [303].

Comparable patterns have been observed in tomato, where inoculation with *B. bassiana*, *M. brunneum*, or *T. harzianum* resulted in relatively minor quantitative changes in volatile emissions but distinct behavioral outcomes. *Metarhizium brunneum* deterred the zoophytophagous mirid *Nesidiocoris tenuis* without affecting the beneficial predator *Macrolophus pygmaeus* [304]. In contrast, *B. bassiana* increased emissions of β-caryophyllene, a compound associated with both pest deterrence and attraction of natural enemies [305,306]. Furthermore, although *T. harzianum* alone did not alter VOC emissions, aphid infestation on colonized plants enhanced attraction of the parasitoid *Aphidius ervi*, likely through herbivory-induced increases in methyl salicylate and β-caryophyllene, compounds known to attract syrphid flies, green lacewings, and ladybirds [307,308,309]. Although the available evidence has primarily focused on VOC-mediated mechanisms, the observed behavioral responses may also be influenced by other plant- and herbivore-mediated factors that remain poorly understood.

Colonization of tomato plants by EEPF significantly reduced the attraction of *P. absoluta* females while enhancing the attraction of the parasitoid wasp *Trichogramma chilonis*. (E)-β-caryophyllene and β-phellandrene were repellent to *P. absoluta* while attractive to *T. chilonis*. Similarly, colonization of tomato (*Solanum lycopersicum* and *S. habrochaites*) reduced oviposition by herbivores, consistent with repellent VOCs, while simultaneously increasing attraction of the predator *Macrolophus basicornis* [124]. Importantly, these effects persisted and were stronger even when the plants were subjected to herbivory to produce higher levels of VOCs. Previous studies have explored the effect of EEPF on insect behavior, such as *B. bassiana* treatment to faba bean seeds, which altered the choice preferences and development of the aphid parasitoid, *Aphidius colemani* [217].

These findings indicate that EEPF-mediated effects on natural enemy behavior are primarily driven by plant-mediated chemical cues and are highly species-specific, depending on fungal identity, plant host, and herbivore presence.

### 4.2. Compatibility of EEPF with Natural Enemies

The compatibility of several species of EEPF with other biological control agents, especially the associated natural enemies of targeted pests, such as predators [310] and parasitoids [80,289,311], has been reported in several systems In many cases, EEPF suppress pest populations without adversely affecting predators or parasitoids; however, these interactions vary among fungal species, host plants, and natural enemy taxa, indicating that compatibility should be evaluated on a case-by-case basis. For example, colonization of sweet pepper by *B. bassiana* and *M. brunneum* reduced *M. persicae* populations without altering key fitness parameters of the parasitoid *A. colemani*, including mummification rate, emergence, development time, sex ratio, and longevity [80]. Similarly, *B. bassiana* did not impair the predator *C. carnea*, which maintained prey consumption rates and showed a preference for aphids from EEPF-colonized plants [98,191]. Comparable neutrality has been reported for parasitoids such as *Phaedrotoma scabriventris* and *Diglyphus isaea* on EEPF-colonized *V. faba* [311].

In some systems, EEPF enhance the attraction and efficacy of natural enemies. Increased recruitment of predators (*M. basicornis*) to *M. robertsii*-inoculated tomato and parasitoids (*C. flavipes*) to herbivore-infested, inoculated sugarcane has been linked with EEPF-induced changes in plant volatile emissions [102,124]. These indirect plant-mediated effects may strengthen top-down control.

However, compatibility is not universal. Behavioral avoidance has been observed in certain multitrophic systems; for instance, *Hyposoter didymator* preferentially parasitized hosts from non-colonized plants, resulting in reduced parasitism of *S. littoralis* on *M. brunneum*-colonized plants, despite successful development of both parasitoid and fungus within the same host [272,312]. In addition, minor reductions in parasitoid emergence or survival have been reported in some cases, indicating isolate- and context-dependent effects [311]. Compelling evidence indicates that EEPF can act synergistically with arthropod predators by coupling bottom-up and top-down processes. Endophyte colonization reduces herbivore performance, increasing prey susceptibility, while predators exert direct population suppression. For example, *M. anisopliae* reduced life-table parameters of *T. urticae* via plant-mediated effects, whereas its predator *Phytoseiulus persimilis* maintained performance and exhibited elevated predation rates and preference for prey from colonized plants [81]. Similarly, endophytic *M. brunneum* reduced fecundity and survival of *A. gossypii* on melon, and its combination with *C. carnea* resulted in synergistic increases in aphid mortality [82]. These findings highlight the potential for enhanced pest regulation through integrated, multitrophic interactions [313].

Overall, EEPF are largely compatible with natural enemies and can enhance indirect plant defenses, contributing to more resilient pest management systems. However, outcomes are system-specific and influenced by fungal isolate, host plant, and ecological context. Rigorous evaluation of multitrophic interactions is therefore essential for effective integration of EEPF into IPM programs.

### 4.3. Potential Risks of EEPF Usage and Non-Target Effects

Although EEPF are generally considered safe, some studies report negative effects on beneficial arthropods, mainly under direct exposure and high doses [314]. For example, *B. bassiana* reduced survival of the coccinellid predator *Coccinella septempunctata* and the lacewing *C. carnea* [315], while *Metarhizium* spp. and *L. lecanii* showed pathogenicity toward the predator *Menochilus sexmaculatus* [316] and lacewing larvae [317], respectively. Similarly, *B. bassiana*, *A. muscarius*, and *C. fumosorosea* decreased survival of *Orius insidiosus*, although fecundity and searching behavior remained unaffected [318]. *Beauveria bassiana* negatively affected the predatory mites *Phytoseiulus persimilis* [41,319] and *Neoseiulus californicus* [320].

In contrast, some natural enemies show little or no susceptibility. For instance, the predatory mite *Anystis baccarum* and lacewing *C. lucasina* were unaffected by *B. bassiana* strains GHA and ATCC 74040, respectively [321,322]. Moreover, combining EPF with predators can enhance pest control: *Menochilus sexmaculatus* improved the spread of *M. anisopliae*, significantly reducing aphid populations, without altering its foraging or oviposition behavior [319].

Behavioral responses also vary. Predators like lacewings and ladybird beetles often avoid fungus-infected prey, likely due to reduced nutritional value or fungal cues, whereas some parasitoids (e.g., *Aphidius* spp., *Encarsia* spp.) maintain parasitism rates despite EPF presence [313,323,324]. In some instances, synergy between EPF and parasitoids has been observed, mostly due to the fact that the fungal strain is more virulent for the pest than the parasitoids, as reported for *C. javanica* (GZQ-1 strain), which was more virulent for the Asian citrus psyllid, *Diaphorina citri*, than its parasitoid *Tamarixia radiata* [313]. Releasing *T. radiata* within 6–12 days after spraying *C. javanica* significantly reduced the *D. citri* population [313].

Additionally, secondary metabolites produced by EEPF may move through trophic levels, potentially affecting herbivores and their natural enemies [325]. Concerns have also been raised regarding persistence of fungal propagules and potential impacts on non-target organisms such as pollinators and soil fauna [326,327,328,329]. Although many risk assessments report limited effects on non-target organisms, including pollinators, predators, and parasitoids, adverse effects have been documented in some organisms and exposure scenarios, particularly following direct exposure. Consequently, the nature and extent of non-target impacts may vary depending on the fungal isolate, target organism, and application method [6,326].

## 5. Constraints and Limitations of EEPF-Mediated Plant Protection

EEPF have attracted considerable attention because of their ability to modify plant physiology and chemistry, thereby influencing herbivore performance, behavior, and multitrophic interactions. Despite these promising attributes, their application as plant-mediated pest management agents remains constrained by several biological, ecological, and technical factors.

One of the principal limitations is the inconsistency of endophytic colonization. Successful establishment and persistence of EEPF are influenced by fungal species and strain, host plant genotype, inoculation method, plant developmental stage, tissue specificity, and environmental conditions [20,32,78,330,331]. Consequently, colonization success and the resulting plant-mediated effects often vary considerably among experimental systems, limiting reproducibility and the transferability of findings across crops and production systems [332,333].

Current understanding of the mechanisms underlying EEPF-mediated pest suppression also remains incomplete. Although fungal colonization frequently alters plant defense signaling, secondary metabolite production, and VOC emissions, causal relationships between these physiological changes and herbivore responses have only been demonstrated in a limited number of systems. Likewise, the relative contributions of plant-mediated defenses, direct fungal interactions, and fungal-derived secondary metabolites remain uncertain because the occurrence, translocation, and persistence of fungal metabolites within plant tissues have not been fully characterized [42,88,334].

Another important constraint relates to the translation of laboratory findings into field applications. Most studies evaluating EEPF-mediated effects on herbivore behavior, performance, and multitrophic interactions have been conducted under controlled laboratory or greenhouse conditions. However, environmental variability, agricultural practices, native microbial communities, and interactions with multiple herbivore species may substantially influence fungal establishment and efficacy under field conditions [332,333,335].

Interactions with chemical pesticides, biological control agents, agronomic practices, and indigenous microbial communities may influence fungal colonization, persistence, and efficacy, yet these interactions remain insufficiently explored. Likewise, continued evaluation of potential non-target effects will be important to support the safe and reliable deployment of EEPF in agricultural systems [332,333,334].

Collectively, these limitations indicate that although EEPF represent promising plant-mediated biological control agents, their reliable application will depend on improving the consistency of endophytic colonization, elucidating the mechanisms underlying plant-mediated pest suppression, validating their performance under field conditions, and better understanding their interactions with other IPM components and non-target organisms. Addressing these constraints will facilitate the effective and safe integration of EEPF into sustainable and resilient IPMprograms.

## 6. Future Use of EEPF in IPM

EEPF are well positioned to become key components of next-generation IPM, shifting crop protection from reactive, single-target interventions toward proactive, plant-mediated regulation. Unlike conventional biological control agents, EEPF influence pest populations through multiple complementary mechanisms, including activation of plant defenses, modulation of herbivore behavior, and alteration of multitrophic interactions. By reprogramming plant metabolism, EEPF can induce systemic resistance, modify VOC emissions, and alter the accumulation of defensive metabolites, resulting in reduced herbivore performance, disrupted host selection, and altered oviposition behavior. Collectively, these effects highlight the potential of EEPF to contribute to sustainable pest suppression while reducing reliance on synthetic pesticides.

Future IPM frameworks are likely to increasingly exploit these plant-mediated effects. Rather than targeting pests directly, EEPF enable a shift toward plant-centered protection strategies, where crops become less suitable and less detectable to herbivores. This is particularly relevant for piercing–sucking vectors such as *M. persicae* and *B. tabaci,* whose feeding behavior underpins virus transmission. By altering vector probing, phloem ingestion, and movement patterns, EEPF can indirectly reduce transmission efficiency of plant viruses, positioning them as dual-action agents against both pests and diseases. Importantly, emerging evidence suggests that EEPF may also enhance plant tolerance to infection through metabolic reprogramming, mitigating disease severity even when pathogen load is not reduced.

A major future direction lies in the integration of EEPF into ecologically intensified IPM systems, including push–pull strategies, semiochemical-based pest management, and conservation biological control. EEPF-colonized crops can function as a “push” component by deterring pest colonization, while trap crops and attractants concentrate pests for targeted suppression. At the same time, EEPF are broadly compatible with natural enemies and can enhance top-down control by increasing the effectiveness of predators and parasitoids that regulate herbivore populations. These fungi may indirectly strengthen biological control by altering plant signaling and volatile emissions, improving the attraction of beneficial arthropods, and weakening herbivorous insects, thereby increasing their susceptibility to predation and parasitism. Through the combined reinforcement of bottom-up plant defenses and top-down regulation, EEPF contribute to more stable and sustainable pest suppression within agroecosystems.

Technological advances will be critical for accelerating the adoption of EEPF in IPM. Current applications remain constrained by inconsistent endophytic establishment, limited propagule stability, sensitivity to environmental stresses, and relatively high application costs. Future improvements in strain selection, targeted formulations (e.g., UV-protected and stress-tolerant propagules), and optimized delivery systems are expected to enhance fungal persistence, colonization success, and field performance [336]. In parallel, integrating EEPF with other beneficial microorganisms, including plant growth-promoting rhizobacteria, mycorrhizal fungi, and *Trichoderma* spp., may facilitate the development of multifunctional microbial consortia for sustainable crop management.

Future research should prioritize mechanistic studies that link plant metabolic reprogramming with herbivore behavior, multitrophic interactions, and disease epidemiology. Integrating metabolomics, transcriptomics, and functional genomics will improve understanding of the molecular basis of EEPF-mediated plant protection, including the roles of fungal metabolites in systemic resistance, plant growth promotion, and abiotic stress tolerance. Equally important are standardized colonization protocols together with long-term, multi-location field studies to evaluate the consistency, ecological robustness, and practical applicability of EEPF across diverse agroecosystems.

Overall, EEPF represent a promising tool for sustainable agriculture, offering a scalable, multifunctional approach that aligns with the principles of agroecology and climate-resilient crop protection. Their successful integration into IPM will depend on bridging mechanistic understanding with applied innovation, ultimately enabling a transition toward more resilient and environmentally sustainable production systems.

## 7. Concluding Perspective

**Figure 5:**
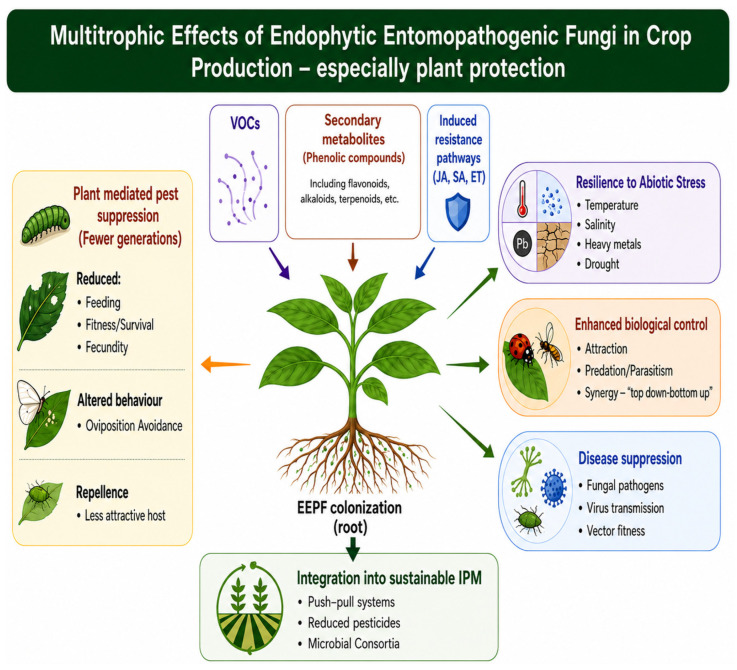
Multitrophic roles of endophytic entomopathogenic fungi (EEPF) in crop protection and their potential in integrated pest management (IPM). Root colonization induces plant defenses (VOCs, secondary metabolites, and JA-, SA-, and ET-mediated pathways), leading to reduced herbivore performance, altered behavior, and increased mortality. Moreover, EEPF also enhance abiotic stress tolerance, suppress plant pathogens, and promote biological control through improved natural enemy activity.

EEPF have emerged as important mediators of plant–arthropod interactions, extending their role beyond direct insect pathogenicity to include plant-mediated defense activation, modulation of herbivore behavior, and the regulation of multitrophic interactions. Collectively, available evidence indicates that EEPF can reduce herbivore survival, delay development, impair reproduction, and alter host selection and oviposition, thereby contributing to plant-mediated pest suppression (Figure 5). However, these responses are highly context-dependent, varying with fungal species and strains, host plant genotype, herbivore identity, colonization success, and environmental conditions.

Current evidence suggests that many of these effects are associated with EEPF-induced changes in plant physiology and chemistry, including defense-related signaling pathways, secondary metabolites, and volatile organic compounds. Nevertheless, the relative contributions of plant-mediated responses, fungal-derived metabolites, and direct fungal interactions remain incompletely understood in many systems. In addition, most available evidence has been generated under laboratory or controlled-environment conditions, highlighting the need for field validation and a better understanding of the ecological consistency of EEPF-mediated effects.

Overall, EEPF represent a promising component of sustainable IPM because of their capacity to simultaneously influence herbivore performance, plant defense, and interactions with natural enemies. Their multifunctional nature provides opportunities to reduce reliance on synthetic pesticides while enhancing crop resilience and supporting more sustainable crop protection. Realizing this potential will require continued integration of mechanistic research with field-based validation to facilitate the reliable incorporation of EEPF into diversified and climate-resilient agricultural systems.

## Data Availability

No new data were created or analyzed in this study. Data sharing is not applicable to this article.
